# “The magic triangle between bed, office, couch”: a qualitative exploration of job demands, resources, coping, and the role of leadership in remote work during the COVID-19 pandemic

**DOI:** 10.1186/s12889-024-17995-z

**Published:** 2024-02-15

**Authors:** Elisabeth Rohwer, Volker Harth, Stefanie Mache

**Affiliations:** Institute for Occupational and Maritime Medicine (ZfAM), University Medical Center Hamburg-Eppendorf (UKE), Hamburg, Germany

**Keywords:** Job demands, Resources, Coping, Leadership, Remote work, Work from home, Coronavirus, Interview study, Qualitative analysis

## Abstract

**Background:**

The COVID-19 pandemic has necessitated many employees to work from home with immediate effect for several months, regardless of their workplace preference or situation at home. Against this backdrop, this study explores perceived job demands and resources as well as the role of leadership and coping strategies of employees and managers with little or no prior experience with working from home in the altered work environment.

**Methods:**

Based on the job demands-resources model, we developed an interview guide and conducted thirty-four semi-structured interviews. The interviews were transcribed and analyzed deductively-inductively using qualitative content analysis.

**Results:**

Experienced job demands include, e.g., challenging, insufficient digital communication, and lack of social exchange, while greater flexibility and work-life balance were identified as valuable resources. Regarding the role of leadership, signaling trust, keeping regular contact, and supporting employees are important. To cope with the unforeseen yet persistent work situation, participants applied creative strategies by setting up offices at home with what they had at disposal. Differences were observed between employee and managerial perceptions as well as over time during the pandemic.

**Conclusions:**

The results expand our knowledge about healthy remote work by adding specific demands, resources, and coping strategies employees and managers experienced during the extreme situation of the COVID-19 pandemic to the picture as well as specifying the role of leadership. Moreover, our findings provide a foundation for guidelines for healthy remote work design and collaboration in times of abrupt change and crises.

**Supplementary Information:**

The online version contains supplementary material available at 10.1186/s12889-024-17995-z.

## Introduction

With the spread of the coronavirus disease (COVID-19) pandemic, the world of work has undergone an unforeseen change. The COVID-19 pandemic urged employees to work from home as part of the infection control measures to contain the spread of the pandemic. In Germany, however, remote work was largely unestablished before the pandemic [1, 2]. Many companies were characterized by a culture of presence at the office [3, 4] and thus little prepared to change the way of working in the wake of the pandemic. Employees had to adjust to their immediate new work situation [5]. This ad hoc adjustment process as well as a different work environment at home represent a change in job demands and resources [6]. Temporary closures or restricted services at gyms, restaurants, cultural and educational institutions presented employees additionally with constrained resources in their private lives and posed a challenge particularly for parents working remotely with stay-at-home children [7, 8]. In Germany, the pandemic prompted employees to work from home as much as possible for several months, starting with the first lockdown in March 2020, and reinforced by the stipulation in the SARS-CoV-2 Occupational Health and Safety Ordinance in January 2021 [9]. While voluntariness of telework (such as in the early stage of the pandemic) seems to have a buffering effect on its perceived negative consequences during the pandemic [10], evidence from pre-pandemic context indicates that more involuntary telework is associated with higher strain-based work-to-family conflict [11] and that teleworking has the most beneficial effects when both remote and office work are done proportionately. Moreover, pre-pandemic findings associate high-intensity telecommuting with increased feelings of isolation, decreased job satisfaction, harmed relationships with coworkers and beneficial effects regarding work-family conflicts [12–14].

Considering this fundamental transformation of work during the first weeks of the pandemic, research suggests that job demands and resources have changed. While some resources (decision latitude, variability) increased and several job demands (social stress from customers, emotional dissonance, work interruptions) decreased over time, lacking communication opportunities seem to particularly burden employees [6]. For remotely working employees, the exclusively digital communication led to a lack of social connectedness [15]. The degree of social isolation faced by employees was found to in turn decrease their adjustment to remote work in the early stages of the pandemic [16]. Apart from loneliness or social isolation, ineffective communication, procrastination, and work-home interference or even family-work conflict have been identified as prevailing remote work demands, whereas job autonomy, social support, self-discipline, and self-leadership were discovered as resources during the COVID-19 pandemic [17, 18]. Emerging literature on coping with the demands of pandemic-related mandatory work from home indicates that employees apply different coping strategies and that these strategies change over time [19]. Prior experience with working remotely and higher trait self-control seem to support beneficial coping strategies, e.g., altering their physical work environment or maintaining their office routine by scheduling their workdays accordingly [20, 21]. As many employees lacked such experience in the beginning of the pandemic [22], managers play an important role to support employee adjustment and well-being working remotely. At the same time, digital collaboration requires different work and leadership practices [23].

Before the COVID-19 pandemic, many managers hesitated to implement remote work practices, fearing a loss of control over employees due to the physical distance [14, 24]. In light of a perceived a decrease in managerial control by both employees and managers during the pandemic [25], a growing body of literature emphasizes the importance of trust in remote collaboration, especially virtual teamwork [26–28]. It is known from the pre-pandemic context that distance has a negative influence on the relationship between leadership contributions and trust in virtual teams, whereas using information and communication technology (ICT) can affect this relationship positively [29]. The unexpected shift from office-based to remote work (and thus, remote leadership) therefore presented many managers with an additional challenge. Both employees and managers perceived their work as generally more challenging during the pandemic. However, managers experienced remote work from home as even more challenging compared to employees, which may be due to additional requirements of remote leadership and collaboration [30] or work intensification [31].

Given this current state of research, we conducted a qualitative interview study to explore the job demands employees and managers experienced, which resources they benefitted from, and strategies they used to cope with these demands. The reasons to follow a qualitative approach are twofold: First, despite existing research on job demands and resources in remote work settings, the unique circumstances of the COVID-19 pandemic (particularly the nationwide lockdown and requirement to work from home) only allow for a very limited transferability of pre-pandemic findings to this specific context [32]. Second, a qualitative approach to examining employees’ and mangers’ individual perceptions is particularly useful as it allows in-depth insights and reveals more nuanced findings through the involvement of the researchers with the participants [33, 34]. Since less is known about *how* people have specifically coped with these job demands during the COVID-19 pandemic and subsequent home confinement [19], given that they only had limited resource at hand, we followed a qualitative approach to reveal more nuanced insights and elicit employees’ and managers’ coping strategies in this situation (see [33]).

We contribute to the literature on maintaining (mental) health in remote work in three main ways: First, we draw on a combination of perspectives by including both employees and managers in our sample. While prior research has mainly focused on employees’ experiences in this context, considerably less research has been conducted on managers’ experiences of remote work during the COVID-19 pandemic [35]. Including managers’ perspective is particularly relevant because perceptions of leadership practices may differ between employees and managers [36] and because leadership can be both, a stressor and resource [37]. It is therefore important to consider not either perspective but both, of employees and managers, to gain a holistic understanding of remote work to derive recommendations for future work design and collaboration. Second, our study focuses on the impact of unexpected long-term remote work due to the ongoing pandemic. In contrast, many studies in this field were conducted in the earlier phase of the pandemic, during the first lockdown (e.g., [20, 38, 39]). In Germany, however, working from home only became obligatory in January 2021, during the third wave of COVID-19 [9]. As a result, the number of employees working from home therefore increased during this period [40], in which we collected our data. Third, we followed an exploratory qualitative approach aiming at gaining a richer understanding of employees’ and managers’ experiences of remote work during the COVID-19 pandemic. We thereby contribute differentiated insights to existing findings by immersing ourselves in the field.

We based our interview guide and allocated our results based on a combination of the job demands-resources (JD-R) model [41, 42] and the transactional stress model [43] as theoretical underpinnings. The JD-R model differentiates between job demands, which are positively associated with strain, and job and personal resources, which are positively related to motivation and buffer detrimental effects of job demands on strain-related outcomes [42]. Since resources are relevant for the processes of appraisal and coping in response to stressors [43, 44], we also explored employees’ and managers’ emotion-focused and problem-focused coping strategies regarding job demands while working from home during the pandemic. The fruitful connection of these theories has already led to enriched versions of occupational psychology frameworks [44, 45] and has been successfully applied in qualitative and quantitative studies before [19, 46]. Hence, we addressed the following research questions:

1. What job demands did employees and managers perceive while working from home?
2. What job and personal resources did employees and managers perceive while working from home?
3. What coping strategies did employees and managers use to deal with the demands they perceived while working from home?
4. What role did managers play in the change process and during the period of remote work from home?

## Materials and methods

### Study design

Following a qualitative approach, we conducted a semi-structured interview study. Since our research questions aimed at comprehending the working situation of employees and managers in a problem-oriented manner, problem-centered interviews were conducted [47]. In problem-centered interviews, prior knowledge can be used to develop interview guides that serve a control and introductory function for interview topics and still allow for openness [47]. Consequently, an interview guide was developed in German language based on the current state of research and the JD-R model [41, 42]. The interview guide initially addressed general information concerning the participants job, experience, and work environment at home as warm-up questions, followed by the main topics of job demands, resources, coping strategies and the role of leadership as well as further questions, e.g., regarding perceived strains, support needs and desires for future work design, which are presented elsewhere [48]. Employees without managerial responsibility were asked about their direct superiors, while managers reflected on their own role as leaders. The interview guide was piloted but required no major revisions.

### Data collection

Data collection took place between May and July 2021, towards the end of the first period of obligatory work from home in Germany. The target group was German speaking employees (≥ 18 years old), who had little or no experience working from home prior to the pandemic, worked more than half of their working hours from home, and had at least six months of experience with pandemic-related work from home. Study participation was promoted at an event of a German business association as well as in their email newsletter and on professional network platforms. Participants were further recruited through professional and private contacts of the authors and their colleagues and based on the snowball system. All interviewees were informed about the background and aim of the study in advance. They were approached by email or telephone to arrange interview appointments and provided with an information sheet on the background of the study and a data protection declaration which they had to sign. Participation was voluntary and participants received no incentives. All interviews were conducted by the first author, who had prior experience in conducting semi-structured interviews. The interviews were conducted one-on-one on the phone or (at the express request of participants) using videoconferencing software. The average interview duration (excluding introduction and debriefing) was almost 30 minutes (± 5.80 min.; range = 18-44 min.). Questions were asked or rephrased only when necessary to elicit further information. All interviews were recorded as audio files. Additionally, the interviewer took field notes immediately after each interview. Interviews were conducted until the authors perceived data saturation was achieved. The audio recordings of the interviews were transcribed verbatim following the rules by Kuckartz [49]. Transcripts were checked for accuracy and anonymity by the first author and not returned to the participants.

### Data analysis

The data analysis was performed using qualitative content analysis with the MAXQDA 2012 [50] software in an deductive-inductive approach. Central to this method is the systematic and rule-governed approach to developing the coding scheme, which was created and differentiated in an iterative process [51]. The coding scheme was based on the interview guide and the theoretical underpinning of this study [41–43]. Sub-categories of demands and resources were allocated to categories of the Joint German Occupational Safety and Health Strategy [52], sub-categories of coping strategies according to the model’s differentiation of problem- and emotion-focused strategies [43], created and differentiated inductively. To ensure data trustworthiness in terms of inter-coder reliability, the coding scheme, initially created by the first author, was tested independently by two members of the research team, discussed and revised until intercoder agreement was reached. The final coding of the interview transcripts was conducted by the first author. Further reduction of the data as well as analyses of descriptive statistics of sociodemographic data were conducted using Microsoft Excel 365 [53].

## Results

### Participants

A total of 34 interviews were conducted and analyzed. 53% of the participants were female. The participants’ mean age was 38.71 years (± 11.53 years; range = 21-61 years), and their mean work experience was 16.57 years (± 13.37 years; range = 1.5-43 years). Most of the participants (94%) worked full-time. More than one third (38%) had no prior experience with working from home and the majority (82%) started working from home directly at the beginning of the pandemic in Germany in March 2020. Regarding their work environments, nineteen interviewees (56%) reported to work in a separate room or workspace in their homes, ten participants (29%) were alone, twenty-three (68%) had partners or other adults present, and four (12%) had children staying at home while working from home. More sociodemographic characteristics of the participants are displayed in Table 1.

**Table 1:** Sociodemographic characteristics of study participants (*N* = 34)

**Characteristics**	***n***	**%**
Gender		
Female	18	53
Male	16	47
Age group (years)		
18-25	2	6
26-35	15	44
36-45	7	21
46-55	6	18
56-65	4	12
Education		
Middle school or completed vocational training	13	38
High school	4	12
University (of applied sciences) degree	17	50
Industry		
Information Technology (IT)	15	44
Manufacturing	2	6
Trade	3	9
Service	14	41
Employment		
Full-time	32	94
Part-time	2	6
Managerial responsibility		
Yes	15	44
No	19	56
Previous experience with remote work		
Sporadic	21	62
None	13	38

### Demands

Employees’ and managers’ perceived demands were allocated to the five categories of the Joint German Occupational Safety and Health Strategy [54], complemented by an additional category for further demands. An overview of the identified demands as well as interview quotes for each category are provided in the Additional file 1.

#### Work content

During the early stage of the pandemic, one employee described the deployment planning as challenging due to the ad hoc implementation of remote work. Another employee described that dealing with customers was difficult from home when his supervisor was not available, and he had to make decision and deescalate conflicts. Serving customers abroad and visiting them on site was not possible due to travel restrictions. These business trips were missed by one employee, who said:

*"Exactly, my customer, for example, is based in the UK. So, I was used to at least having video calls with them, so nothing has changed at all. And otherwise, so what I just miss is seeing my colleagues at work and we just used to have team events and I was also at the customer's a couple of times a year and stuff like that is of course missing, but how the company is supposed to improve that now, yeah, that's difficult." (employee #7, female)*

#### Work organization

Regarding work organization, employees and managers stated that when starting to work from home, they felt rather lonely at the workplace. A manager referred to the change process regarding digital work organization within the own organization but also when working with clients as challenging. One employee also found additional digital formats to facilitate remote exchange among colleagues stressful. Another manager described digital instruction during the early stage of remote work as exhausting because digital solutions had not been established yet. Communicating digitally was perceived as demanding both initially but also throughout the period of working from home. In the beginning, it was unfamiliar and difficult due to lack of digital skills. For managers, it required planning and made support and exchange with external partners more difficult. It was especially perceived to increase the inhibition threshold for communicating with colleagues. Throughout the pandemic, digital communication was found not to be able to keep up with face-to-face communication. It partly led to misunderstandings, changed social interaction, trust, and discussion culture. It also impeded creative collaboration, increased workload for managers, made instructions, rhetoric, recognizing reactions and getting to know new colleagues more difficult. In sum, digital communication could not replace face-to-face communication and collaboration. Both employees and managers also mentioned that digital communication required more effort and thus made work more demanding. Not knowing if or where a colleague is working also inhibited contact. Communication seemed less direct but rather purpose-bound and formalized. Moreover, employees and managers described the lack of (professional) exchange as demanding, in the beginning but also afterwards. This was especially due to the lack of joint presence in the office. Employees and managers reported persistent difficulties in complying with working hours and taking breaks. Due to the remote work regulation, employees and managers were not able to meet new colleagues in person. This lack of face-to-face meetings also impeded proactive communication. Remote work and not meeting colleagues on site thus led to a high frequency of video and phone calls for employees and managers. However, these calls were not necessarily perceived as useful but rather straining, causing psychological and physical symptoms, leaving no time for breaks or other tasks. Despite this, managers mentioned that their prior commute time was now missing to make phone calls and achieve their objectives. Employees described that vocational training was more complicated working remotely because they need to be trained in the office and as intended by law. Moreover, remote work, due to physical distance, was perceived to impede the flow of information among employees and managers, e.g., to generate new orders:

*"So, we sit here [in the office] in one room on purpose. The communication is extremely high. And, if one person is on the phone, the rest of the people hear that and then we're just like, ‘Where was that now?’ ‘Yeah, that was there and there.’ ‘Ah, all right.’ And then you're informed. That’s all eliminated now." (manager #2, male)*

#### Social relationships

During the early stage of remote work, being new was described as particularly challenging. Managers ascribed this to having little contact with their team members and the need to build trust. Employees attributed this demand to digital onboarding, the difficulty to acquire knowledge, and not knowing colleagues personally. Thus, new employees sometimes only knew the names of their colleagues but were not yet able to establish more personal social relationships with them. An employee and even a manager stated that they did not initially receive support from their supervisors, at least not needs-specific support. From the employee perspective, little contact with their supervisors, especially receiving little feedback and feeling left alone, were mentioned. The most frequently reported demand concerning social relationships, throughout every stage of working from home during the pandemic, was the lack of contact and private exchange among colleagues. Employees and managers attributed this to the lack of common breaks spent together or other on-site encounters in the office, the higher inhibition to make digital contact, digital communication being mainly work-related and not able to replace face-to-face conversations, but also to new team affiliations or when being new to the organization. It made team building more difficult und was further perceived to change closeness to team members and impair the team spirit. One employee mentioned conflicts among colleagues about who was allowed to stay in the office or work from home. Also, it led to less social interaction during and after work, and feelings of loneliness. Thus, a manager emphasized the perceived deterioration of team spirit due to a new team structure which was established during the pandemic. He referred to the challenges of digital communication in this context:

*"So, the will to turn on a camera, an order is doubtful from my point of view. Is not necessarily purposeful. I have felt a clear deterioration of the team structure, clearly. So, the team cohesion was very strong before. Due to the organizational change, the team has of course been reorganized, some have joined, some have left, that was of course an aspect. But overall, the team structure has deteriorated considerably. And we were able to hide a little further away.” (manager #8, male)*

#### Work environment

Regarding the work environment at home, the (lacking) procurement of work equipment initially created a challenge for a manager. Working in the IT service industry, he highlighted difficulties in obtaining well-functioning and affordable hardware due to shortages at the beginning of the pandemic due to widespread implementation of remote work. Initially, but also throughout the pandemic, employees and managers could not access work materials they needed in some cases, e.g., because it required access to network drives or analog original signatures on documents. Taking care of this complicated the exchange with external partners and brought additional organizational effort and thus caused a higher workload. Most statements regarding work environment-related demands referred to insufficient work equipment at home. Managers and employees lacked hardware including monitors, video cameras, headsets, and printer, required settings but also ergonomic furniture (desk, chair) and sufficient lighting. They further named slow internet connection at home and inadequate IT provided by the company. Many employees and managers were not alone when working from home. Oftentimes, their partner worked from home as well or other housemates or children were present. Some interviewees even shared one room or workspace at home or had to deal with parallel family demands. A few reported it impacted their relationship, required room arrangements, or led to conflicts with their partner. Overall, many interviewees described the presence of others while working at home as demanding:

*"That's why in this case and by the fact that I still had the influence children on site here, it was a very unpleasant experience for me. On the one hand, that has to do with concentration, focused work, and on the other hand, that actually has to do with the leadership activity, because I felt uncomfortable and not good with leadership at a distance." (manager #8, female)*

#### New forms of work

Especially after the ad hoc changeover when beginning to work from home, some employees and managers suffered from their lack of experience with working from home. It necessitated interviewees to reorganize their everyday lives. Employees also reported they initially felt insecure about digital communication. Mangers further highlighted their uncertainty about legal aspects of remote work at home, its technical implementation, and employees’ personal requisites. Managers further elaborated on the challenges of digital leadership, which they mentioned as demanding during the early stages but also throughout the whole time of remote work. Initially, some were uncertain whether employees would actually work at home and how to reach them emotionally by digital means. The ad hoc loss of proximity and difficulty of team building contributed to this feeling as well as a general discomfort with digital leadership. As consistently perceived demands of digital leadership, managers stated that it was not perceived suitable for personnel conversations. It generally required more time and managers feared they could not fulfill their duty of care well enough, e.g., because emotions were not evident, and it was difficult to convey motivation at distance. The greatest demand regarding this new way of work can be summarized as the lack of spatial separation of work and private life. Employees and managers found it more difficult to mentally switch off from work during leisure time or to call it a day in the evening due to the constant availability and accessibility of work equipment, as well as due to lack of spatial separation of work and leisure. The lockdown, which restricted recreational activities, also contributed to this. However, in view of all these job demands while working at home during the pandemic, an employee and a manager stated they perceived work in the office easier when returning on site was possible. Some employees talked about having a bad conscience about not working at home. Having longer conversations with colleagues was suddenly not perceived as working time anymore in contrast to having such conversations on site in the office:

*"[…] you watch yourself and say, 'Gee, now I've been chatting with him for so long again,' and in the home office you also feel a certain obligation not to go overboard, so that you don't make a bad impression working from home. […] But if you transfer it to your work, what was it like in the past? Then you sometimes talked just as long, but then the boss probably even joined in and chatted for a while and then you didn't have this, you didn't have this bad feeling about it, you have to say quite clearly." (employee #12, male)*

#### Further demands

Further demands participants mentioned referred to the consequences of the pandemic and related remote work. For example, leisure activities and thus, recreational opportunities, were restricted. This affected both employees and managers throughout the time spent working from home. It was even aggravated by the second lockdown during the winter season 2020/2021, when the incidence reached a new peak. Throughout the pandemic, the increased use of digital media for leisure activities was described as another demand. Both employees and managers mentioned the need to cook for themselves at home was another time-consuming stressor. At the beginning of the pandemic, uncertainty about unforeseeable duration and consequences of the pandemic burdened employees and managers. Even over the course of time, the lack of clarity about remote work regulations burdened some employees. In particular, the differentiation between (contractually agreed) telework and (not legally defined) mobile work in Germany was mentioned here, as many companies implemented the pandemic regulations in such a way that employees were responsible for their work arrangements. Over time, as office work became possible again, one employee reported that she found office work to be less productive after having worked from home:

*"But even now I have to say that I was only in the office last week and when I compare that, I get a lot less done. Simply because you're excited to see your colleagues again, you're chatting away again. So, I think this focus has simply shifted. What was perhaps normal in the past, standing by the coffee machine and having a chat here and there, is simply no longer something you're used to. At home, you just work all the time, you might not have that exchange either, and now you perceive that as being unproductive when you're in the office, actually." (employee #2, female)*

### Resources

Employees’ and managers’ perceived resources were allocated to the five categories of the Joint German Occupational Safety and Health Strategy [54], complemented by an additional category for personal resources as intended by the theoretical framework [41, 42]. An overview of the identified resources and further interview quotes are provided in the Additional file 1.

#### Work content

Generally, employees emphasized that enjoying their job helped them to cope with the unusual circumstances. Managers, on the other hand, appreciated the elimination of business trips. One manager also highlighted that the company – as part of the IT industry – actually benefitted from the pandemic. At the beginning of the pandemic, one employee and one manager felt their job-related participation in the process of moving work from the office to home was particularly helpful in adjusting to this change:

*"So, I even have a different view than just: I've been transitioned, and I've been working from home. I was also involved in making it possible for all people in our company to work from home. Because our IT department made sure that within a week, and we received a lot of praise for this, all of our employees were able to work from home. And the changeover was surprisingly relatively harmless for me […]" (employee #4, male)*

#### Work organization

Both employees and managers described the quick implementation of home office arrangements at the beginning of the pandemic as helpful. One employee particularly referred to the rapid provision of work equipment in the home office. Managers rather emphasized daily communication during this early stage and benefitting from considering employee experiences for technology procurement and having switched from stationary computers to laptops recently. Both during the early stage of the pandemic as well as throughout the period, employees, and (in the latter case) managers reported fewer disturbances when working from home. This was primarily attributed to a quieter work environment and fewer distractions compared to the office, which allowed for more focused work. While working from home during the pandemic, employees also described being more conscious about taking breaks. Setting automatic reminders helped to take breaks and not being available and changing locations during breaks made them more relaxing. One employee mentioned the working time recording system at his company, which helped him to comply with working hours and to separate work and leisure. Employees and managers also highlighted the benefits of digital communication. Employees pointed to the avoidance of loneliness and the ability to stay in touch with colleagues, identify their availability, and enable collaboration. Managers emphasized that accessibility to colleagues was improved, collaboration was simplified, and the number of conversations was reduced due to a higher barrier to being contacted. Digital communication also allowed them to stay in touch with colleagues and attend more (online) meetings, as well as create (emotional) distance when needed. However, it also allowed to participate in more appointments:

*"On the other hand, the fact that I am responsible for two regions means that I am, and I don't mind admitting it, sometimes in conference calls or phone calls at the same time. That's an advantage of home office, of corona, because when I'm present somewhere, I don't go to another conference call, well then you leave sometimes. But then I can use that a bit more in these corona times. Of course, that's not always so ideal, but well, I weigh it up and the chances outweigh now and then to be somewhere at the same time. Because I don't have to move spatially, I can do everything from my desk." (manager #7, male)*

#### Social relationships

Regardless of the phase of the pandemic, peer and supervisor support was highlighted as an important resource by both employees and managers. In addition, both employees and managers particularly appreciated seeing their colleagues on office days whenever possible. Employees also emphasized that they have regular contact with their supervisors, especially during regular appointments. Another resource at the social level throughout the pandemic was strong team cohesion. This resulted in part from overcoming the lows and challenges of the pandemic together. Moreover, (digital) team events such as virtual Christmas parties helped maintain team cohesion. However, only one employee and one manager said they had this experience. Both employees and managers emphasized the importance of trust for appreciation, motivation, job satisfaction and cooperation. At the beginning of the pandemic, however, one employee particularly benefited from the support of her colleagues. During the pandemic, one manager noticed a change in his team. It seemed that remote collaboration led to more equality among international team members:

*"[…] now, when we have a big team meeting across the markets - before it was just the German team sitting in an office and the rest of the teams somehow joined in via [Microsoft] Teams - suddenly everyone was the same. So, no one was close to the boss, I would say, because everyone worked virtually, and that led to a bit of a convergence, at least between the teams in Austria, Poland and Switzerland. To me and to the whole team structure." (manager #3, male)*

#### Work environment

At the beginning of the pandemic, employees and managers benefited particularly from the provision of work equipment by their employers. Some employees were able to simply take the devices home, others were fully equipped by their employers or supported in the procurement of hardware. Still, others supplemented their provided work equipment with private devices. Managers also mentioned the possibility of taking devices home from their offices and receiving support from their employers. One employee appreciated the rapid availability of IT support during this early phase of the pandemic when problems arose. In this early phase, as throughout the pandemic, employees and managers stressed the importance of being well equipped in the home office. In particular, they pointed out that the appropriate equipment was already available at home, or that the existing equipment could still be improved (ergonomics and well-working IT). While the importance of good work equipment and environment at home was highlighted by employees and managers regardless of the phase of the pandemic, one employee and several managers reported having better equipment at home only after some time because it became available later or because IT got better over time. Employees and managers also enjoyed being able to work standing up in their home offices. Furthermore, companies’ financial and material support for the procurement of work equipment during the pandemic was appreciated by employees and managers. Digital collaboration tools were also highlighted by both employees and managers, e.g., to keep track of projects or to stay connected. In addition, one manager stated that also the pandemic-driven implementation of digital documents facilitated the collaboration with contractors. Forced by the external circumstances, they digitized processes, making collaboration more efficient regardless of the pandemic. Those employees who were used to desk-sharing systems in their offices especially appreciated having their workspace at home right at their fingertips without having to set it up again each morning:

*"[…] we have these setup times, this desk sharing. That means we have to set up our laptop every day at the workstation, connect it to the monitors there, with a separate keyboard and so on. For hygienic reasons. And that also takes up a bit of time. In the worst case, you have to get a small trolley out of a small safe, where your laptop and everything you need is in it, then you look for the workstation, which you have to book in advance, then you work there, of course you have to set everything up beforehand, wire everything, connect the monitors to the docking station, the mouse, keyboard, webcam if necessary, the headset and you haven't seen it. And then you work and then it's the same thing backwards again. That's just such a little thing, of course, but that's an incredibly noticeable process because that just sucks. It's really just a total little thing and there are really worse things, but it's one thing that really gets on a lot of colleagues' nerves because it's also so ineffective." (employee #12, male)*

#### New forms of work

From the beginning of the pandemic, those employees and managers who already had experience with digital work benefited from this. This was partly because they were already used to video conferencing, collaborating by phone or digital leadership, were part of cross-site teams, or already had experience working from home or other locations. Likewise, employees and managers enjoyed being able to organize their day more freely, choose comfortable clothing, and be subject to less scrutiny. Saving time was mentioned by almost all interviewees. It mainly resulted from not having to commute to the office or to set up their workstation in the office. As a result, employees and managers were able to spend more time with their partner, family or pets, had more free time or time for themselves, and were able to sleep longer. Finally, lower fuel costs due to the elimination of commutes and the ability to even sell a vehicle resulted in a financial windfall for some employees. Over the course of the pandemic, many employees and managers said they had become accustomed to working digitally. They learned to separate work and personal life while working from home and how to use digital media, improved their self-organization, and established or changed their routines where necessary. Another change that one employee experienced was greater spatial flexibility in terms of work location. It also helped them integrate work and personal life, such as completing household tasks, attending personal appointments, accepting packages at home, balancing work and continuing education or childcare, as well as creating more free time and integrating sports and exercise into their workday. Some employees and managers managed to keep work and free time well separated. Some did not find it stressful to mix both life domains, others made agreements with superiors and had a clear understanding of the evening as free time, which helped them to achieve this. In one case, a merger of the company allowed a participant to choose new office locations even beyond the office and the home. Irrespective of the pandemic phase, temporal and spatial flexibility of work were appreciated by several employees and managers:

*"[...] But I realize that this way I find a good mode between: Hey, I'll take a break for an hour at lunchtime and then check in again in the evening or answer quickly. Or, if I have a creative phase on a Sunday afternoon, because it's just quiet and I come up with a few ideas for the concept, then I somehow briefly do that again on a Sunday evening. And then I take Thursday afternoon off for that. So that's super flexible." (employee #8, female)*

#### Further resources

At the beginning of the pandemic, one employee stated that her family protected her from isolation. In all phases of the pandemic, employees referred to workplace health promotion offerings. These offerings, which were mostly recognized and appreciated by respondents but not used, included advice and guidance on ergonomic workplace design, anonymous psychological contact points, exercise tasks and compensatory exercises for the neck and back, health days, and the provision of meals for preparation at home. Throughout the pandemic, children's sick days provided support to parents, especially during the times when kindergartens were closed. Managers highlighted seminar offerings on digital or hybrid leadership that helped them to adjust to the lack of face-to-face contact with employees and care for their staff to better identify how they are doing at home. Caring for pets also helped interviewees to care for themselves (e.g., ensuring to take breaks), as two managers reported in reference to their dogs:

*"Well, I make sure that as far as tasks can be completed, they are completed. And I also finish them on time. But I also have some personal obligations because of my dog, who shoos me out and says: 'I'd like to finish now, please.' ((laughs))" (manager #12, female)*

#### Personal resources

Regarding personal resources, one employee mentioned that particularly at the beginning of the pandemic, experiencing meaningfulness in her job helped to stay motivated alone at home. Also, one employee and one manager mentioned that they were grateful at the beginning of the pandemic for the opportunity to work from home to avoid infection. Later during and throughout the pandemic, an employee benefited from the fact that he was used to sitting at the computer a lot. Accordingly, the change to working from home did not bother him much. One manager indicated that support in her home environment, i.e., from school and family, was a valuable resource for her to combine childcare and working from home. However, the autonomy and flexibility of working from home also required self-discipline, which helped both one employee and some managers work productively. One employee described serenity as a resource for accepting given circumstances instead of letting them drag them down. A manager also mentioned mindfulness as a resource. She started being more mindful while working from home. Both employees and managers reported that self-reflection was an important resource for coping with the circumstances of being alone and responsible for organizing their work and leisure:

*"Well, maybe what I did a little better is that I dealt with myself a little better, because I just spent so much time with myself. Even though you work at the same time, of course, but it's always something else when you work unobserved than when you always have people around you. So, I think you just reflect on yourself a bit more, which I actually think is quite good." (employee #10, female)*

### Coping strategies

Employees’ and managers’ coping strategies were allocated according to the theoretical model [43]. An overview of the coping strategies mentioned and corresponding interview quotes are provided in the Additional file 1.

#### Problem-focused coping strategies

In the beginning of pandemic-related work from home, employees reported that they coped with this new situation by preventing physical damage and making virtual collaboration as easy as possible. One employee began to do compensatory exercises against unhealthy sitting postures at home. Employees and managers also introduced digital exchange formats at work, such as coffee calls, after-works, virtual team events, regular team calls or individual meetings. One employee working in personnel development started to proactively signal her availability and asked colleagues about their well-being. Early on, managers made sure that files would be digitally available to all or built on pre-existing digital tools that they already used. After the initial uncertainty of the pandemic situation, employees and managers resumed their pre-pandemic habits. Also throughout the pandemic, adherence to routines helped employees and managers to establish daily structures, e.g., by pretending to go to work (in the office), wearing the same clothes and keeping the usual working hours from the office when working from home. To comply with working and rest times, some employees and managers scheduled break times, evenings off and fixed private follow-up appointments at the end of work. Various other temporary boundary management strategies also helped employees and managers to mentally switch off during leisure time. For example, one employee regulated his accessibility during leisure time by uninstalling work-related apps from his smartphone during vacation. One manager set a reminder to terminate his workday. In similar vein, employees and managers used spatial boundary management strategies to help them detach from work. They changed locations or rooms at the end of the workday. To this end, some interviewees set up separate workspaces within their homes by converting rooms into home offices or even moving to larger apartments because their partners also worked from home. To support psychological detachment from work, employees and managers began to store their work equipment out of sight or put it away after work. In one case, an employee even transformed her home office/bedroom completely thanks to a foldaway bed and table, which helped her to not feel like still being in her office after working from home:

*"[…] I have a foldaway bed and my table is also so relatively foldable. That means that on most days I really fold everything up after work, put my laptop and keyboard in my work backpack, sometimes fold up the table and then unfold my bed again, so that my room, so to speak, even if it's the same room, looks different during the day than in the evening, which I find to be quite an advantage all the time, because I somehow never have the feeling: 'Wow, I'm living here in my office or the work is coming home with me.' But rather, through such a few moves, the room is restructured after work in such a way that it's no longer an office, but my room." (employee #18, female)*

After a while, employees and managers started to upgrade to work equipment (e.g., by buying swivel chairs or height adjustable desks) after some of them had suffered from musculoskeletal complaints but also for more comfort or to improve their technical equipment. Some employees and managers also found creative solutions to compensate for missing work equipment:

*"So, you sit straight for the first 30 seconds and then you slump and depending on how concentrated you are working or what kind of activities, so I don't know working with an excel sheet on a small screen is of course deadly for your back ((laughs)). When I'm on the phone, it's okay. I also make sure that I stand up. Provisionally. I simply turned a laundry basket upside down and put it on my kitchen counter and put the laptop on it. So, it all kind of works." (manager #14, female)*

However, upgrades and devices were not always helpful. To prevent headaches, one manager mentioned that he had to put down his headset due to the myriad of calls in which he participated daily. Some managers also used private material, especially for postal traffic since they could not come to the office. Those employees and managers who sometimes had the opportunity to tried to go to their offices pooled and completed their tasks which required their presence in the office collectively on these days. Being able to go to the office was also accompanied by arrangements on office use among colleagues. Some made direct agreements, while others established a rotation model or used digital tools to keep track of office presence. One employee planned her tasks according to the opportunity to work in the office. She couldn’t motivate herself to work strategic at home and therefore completed them on her office days, shifting more day-to-day business to the home office. Even within one workday, employees and managers structured prioritized their tasks, and organized deadlines to structure their work and tasks. They used their professional experience in ranking importance of meetings and digital tools for prioritization.

Regarding digital communication while working from home, employees and managers found various coping strategies to make it work. One employee mentioned that they established rules for digital meetings in their team. They included turning on cameras and hearing each other out. Similarly, several other employees and managers emphasized the importance of using richer media such as video cameras to improve contact and collaboration. One manager described that he lacked feedback due to the digital communication with this team. Therefore, he tried to compensate this digitally with anonymous surveys or during online meetings. Not meeting colleagues on site also led to actively seeking exchange among employees and managers. Some managers emphasized the importance of exchange with other trusted managers or close colleagues. Nevertheless, managers also reported that important conversations need to be conducted in person, face-to-face. They especially referred to personnel appraisal meetings with employees and conversations to solve problems among employees:

*"At the moment, we have what I would call an interpersonal problem in one team. And that's very difficult to sort out over the phone or by e-mail. And I have just now, before you called, set a personal appointment for us to sit down together next week and sort out this problem." (manager #11, female)*

### Emotion-focused coping strategies

Emotion-focused coping strategies were mostly reported by employees. During the early stages of pandemic-related work from home, one interviewee searched for new hobbies like learning a new language, taking care of plants, or doing sports. Some of the reported emotion-focused coping strategies only emerged over the course of time, e.g., one employee started to simulate the office environment by turning on the radio. Likewise, employees meditated to maintain emotional balance or cope with loneliness. Another employee mentioned resignation after some time because working from home was very demanding for her. Few managers further mentioned they were more relaxed when handling IT problems because they were getting used to it over time. After some time during and throughout the pandemic, exercise and sport helped employees to cope with their perceived stress. It helped them for better concentration and to compensate for the lack of exercise. Some employees and managers moved during work in breaks or during phone calls, some managers exercised before or after work:

*"[…] when I want to go to the office, I first have to walk 15 minutes from my home to the station and I just did that every day there and back again every day, simply because I walked this way. And that's when I noticed that I was missing the exercise, because even if you don't actively do sports or anything, you move a lot more, simply because of the journey to the office. And I noticed that I was somehow missing that, which is why I decided to simply integrate this sport into my everyday life.” (employee #10, female)*

One employee reported that she mediated conflicts among colleagues through appeasement throughout the pandemic. Another emotion-focused coping strategy that helped her to avoid conflicts was not taking things personally based on written or online communication. Both employees and managers talked to their partners and friends about their feelings and experiences during the pandemic and work from home. Lastly, pursuing hobbies helped employees and managers to compensate demands from pandemic-related work at home. Their hobbies included spending time with pets, meeting friends digitally, reading, watching series, playing computer games, doing sports, riding their bicycles, and gardening:

*"Well, I'm outdoors a lot, but I still try to free up the appropriate time for specific sports, as far as possible due to corona. I'm in the garden and do things that are good for me." (manager #12, female)*

### Role of leadership

Employees’ and managers’ perceptions on the role of leadership were distinguished between leader and follower perspectives. An overview of the identified roles of leadership from both perspectives and interview quotes are provided in the Additional file 1.

#### Employees’ Perspective

From the employees’ perspective, five categories were identified concerning the role of readership: managerial communication, attitudes and behaviors, support provision, staff-care, and trust. For managerial communication, employees mentioned the role of leaders regarding bilateral and team-level communication most frequently. This mainly related to the frequency and regularity of calls, especially in the early stages of pandemic-related remote work. Few employees highlighted the constant availability of and proactive contact establishment by their supervisors:

*“[My supervisor] just calls in between to find out how I am, how things are going. [...] But this active calling [after online meetings] and asking, ‘Can I give you feedback on this? Maybe you can take that into account next time.’ I found that very appreciative [...]” (employee #11, female)*

Regarding managers’ attitudes and behaviors, employees mentioned managers’ endorsement as well as negative attitudes toward remote work, which even led to unfair and unsafe managerial behaviors and determined how remote work regulations were implemented within the organization:

*“From my point of view, my supervisor rested on that because he refused to work from home all the time. And at times I felt that was a bit unfair. Especially during the time when invoices were due, we realized that my colleague and I both had to go to the office, because otherwise there would have been far too many mistakes. Because of this coordination problem [that the three of us share the office]. And even then, our boss was there instead of saying for two days, ‘Well, then I’ll just go home.’” (employee #1, female)*

Other managers endorsed remote work and gave employees clear expectations, responsibility, and autonomy, and encouraged them to ask questions:

*“I somehow also said that I am struggling with calling people because of small things or so. And I have been quickly told [by my manager] that it shouldn’t be that way, and I should simply call, it’s no problem.” (employee #10, female)*

Another important role of leadership was described as providing support. While needs-specific and manager support was sometimes lacking in the beginning of remote work in the pandemic, most employees received support from their supervisors overall, especially regarding work organization and feedback:

*“So, my supervisor has taken care of a lot of things that otherwise would have arisen somehow implicitly. Yes, because on the other hand, of course, being the contact person, not only for technical questions, but also, if something goes wrong, but ultimately also for technical things, my monitor doesn’t want to connect today.” (employee #17, female)*

Managers’ staff-care, referring to inquiries about employee well-being and wishes was also acknowledged by several employees:

*“Well, my manager regularly asked us whether we could balance, but also separate our work and private lives at home, and how we were doing.” (employee #6, female)*

Lastly, employees emphasized the importance of being trusted by their managers, which was perceived to positively influence employees’ satisfaction with remote work:

*“I am pretty satisfied [working at home]. Especially because I was also given feedback by my manager in my feedback meeting that I was doing my tasks well and so on. And that’s why I rather had the feeling that this also has a lot to do with a lot of trust in me and yes.” (employee #18, female)*

#### Managers’ perspective

Managers brought up similar topics regarding their role as leaders. However, they differed in valence compared to the employees’ perspective. Staff-care was mentioned most frequently, followed by the difference of digital leadership, communication, trust, and flexibility. Staff-care, from managers’ perspective, included taking employee health seriously and caring for sufficient work equipment, considering individual differences, paying attention to not lose sight of employees, providing support, absorbing uncertainties but also keeping professional boundaries. This also included rebuking employees to protect their health and private life:

"Some people you just have to make them follow the work rules. ‘Yeah, I know, you're on something right now. You still can't work ten hours a day without justification.’ Or in plain language, ‘F*** off, please.’ ((laughs)) ‘Now is the excellent time to clock you out.’ That's when you have to protect people from themselves a little bit, too." (manager #2, male)

Managers highlighted the special role of digital leadership and its difference compared to face-to-face leadership. Digital communication made leadership difficult for some. It required more time and consideration of privacy, while keeping track of team performance, leading to role conflicts. Few managers initially felt uncomfortable with digital leadership, mainly because they were unsure whether employees would actually work at home. They perceived difficulties motivating them remotely, and lacked feedback from their team members:

*"Because we were always present before and a lot, I'm a manager, I think, who communicates more via symbols or motivation. You can't convey motivation verbally, so you can't do it very well via a Webex or an online meeting, I think. And, since not everyone is always equipped with a camera or has used one, direct contact is missing. So, the facial expressions and gestures of employees are missing, and you don't get that back and have a very bad feeling. You can't really assess where you stand and where the team stands." (manager #8, male)*

Communication as a leader was another topic mentioned by the managers themselves as well. They focused on conducting critical conversations face-to-face and establishing contact proactively. Managers emphasized bilateral and regular talks with employees and incorporating small talk and being transparent in their communication. While digital communication was also perceived as hampering and challenging to find a balance, consistent digital communication was also recognized to align the proximity of team members. They further highlighted their visibility as a leader:

*"[…] when I communicate with the team, I always turn on my video. The fact that I'm also a manager means that people can see me, that they can see that I'm not sitting there with curlers in my hair ((laughs)) and, I don't know, in a dressing gown. And I also think it's very important for employees to see a face, regardless of whether it's a personnel issue or a management issue. For me, that's trust on the one hand, and the other is just, okay, creating understanding." (manager #1, female)*

Trust was attributed to the role of leadership by managers as well. Thus, digital leadership was built on trusting employees that they do work at home and turn to their manager when needed:

*"Otherwise, I don't really like the word, but my leadership style is actually more that you have a lot of trust in each other and that everyone sets it up for themselves the way it works and that, I always like to say, as soon as I don't hear anything, I assume that all is well, but please talk to me if you have a stomachache or if you see an issue somewhere." (manager #14, female)*

Lastly, the role of leaders included finding and providing flexible solutions. They referred to granting employees autonomy and using flexible conversation formats. Moreover, flexible solutions were preferred to allow for remote work at home without major contractual and administrative effort:

*"[…] I still see some legal problems there when I get this [teleworkplace contractually] signed. So, on the one hand, things have been neglected in the draft [of telework regulations], on the other hand, the employees are made worse off and now have certain supervisory duties. And I simply don't want them to perform them at all. Because then, if you really take it literally, there's a paper tiger behind it. And I just said, ‘You know what, you're going to get a chair, if you ever stop working here, please bring it back.’" (manager #2, male)*

**Fig. 1:**
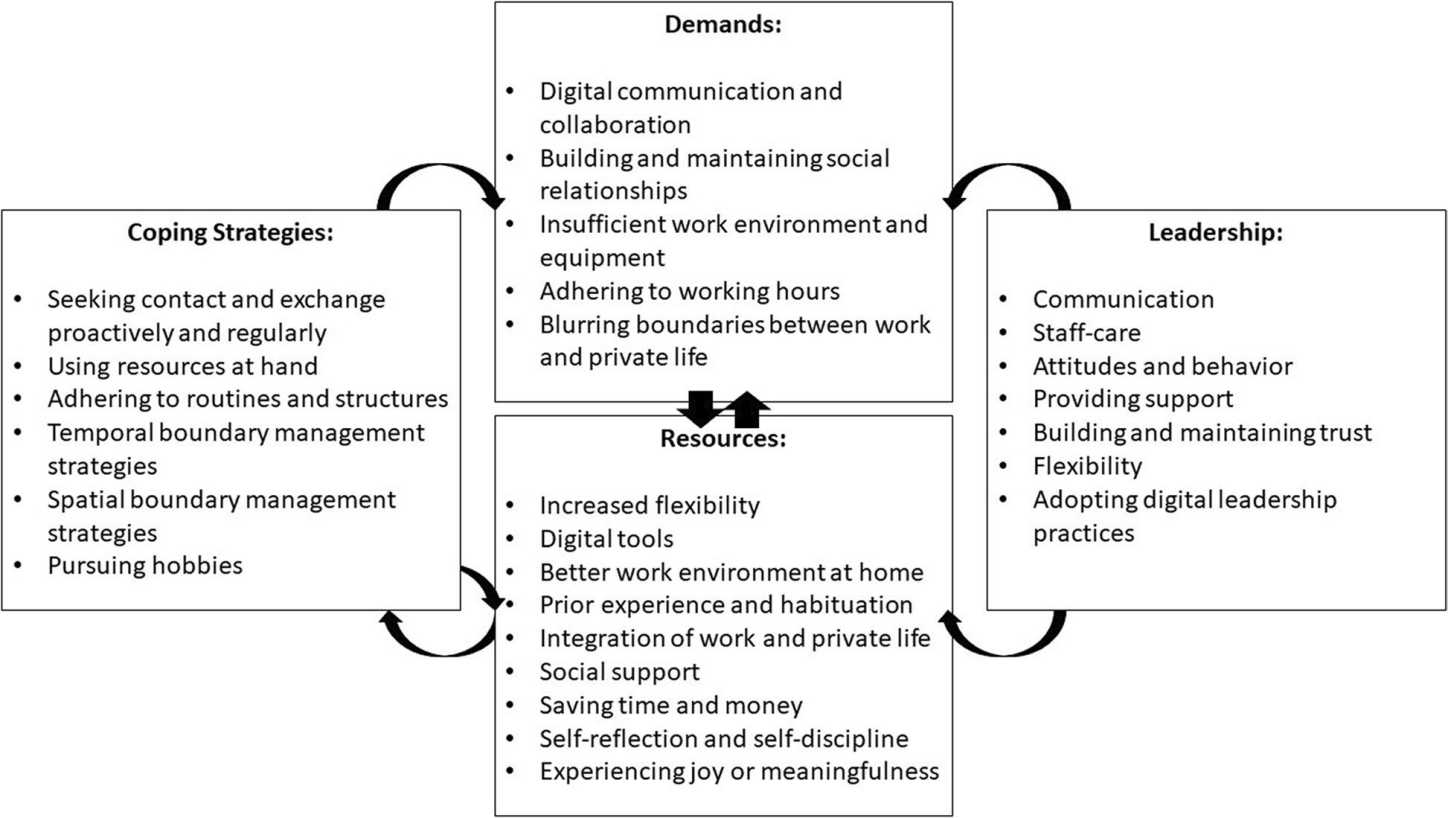
Overview of the interplay of (selected) identified demands, resources, coping strategies, and the role of leadership

As these findings demonstrate, leadership can be (or amplify) a demand as well as a resource (e.g., low managerial support or high staff-care). The interaction of leadership with demands, resources, and coping strategies is depicted in Fig. 1. This conceptual summary of our findings illustrates a selection of the identified demands and resources that may interact (see [41, 42]). Resources enable employees and managers to apply coping strategies that, in return, can leverage new resources and reduce demands.

## Discussion

Building on the theoretical frameworks of the transactional stress model [43] and the JD-R model [41, 42], the aim of our study was to explore employees’ and managers’ experiences of mandatory working from home during the COVID-19 pandemic. More particularly, we investigated their job demands, resources and coping strategies, as well as the role of leadership in semi-structured interviews. Our results contribute profound qualitative findings to the occupational health literature about virtual work and telework in two ways: First, our study provides in-depth insights, such as creative coping strategies with what employees’ and managers’ had at disposal (e.g., using an upside-down laundry basket to be able to work standing up) given the peculiar situation of having to set up an office in their own homes on an ad hoc basis. Second, our results not only enrich, but also provide further empirical support for extant qualitative (e.g., [19, 55–57]) and quantitative findings (e.g., [16, 30, 58]) on working conditions and well-being in remote work during the COVID-19 pandemic.

### Individual perceptions of job demands and resources

Many of our findings on demands and resources referred to the digital communication and cooperation, which have substantially changed with the mandatory shift to working from home. Our results also reflect the diversity of individual boundary management preferences [59] and tactics [60], such as segmentation and integration. The mandatory nature and extent of working from home during the COVID-19 pandemic could lead to a misalignment of personal segmentation preferences and work-nonwork balance [61]. In this situation, employees and managers applied different segmentation strategies (i.e., behavioral, temporal, physical, and communicative) within the scope of their possibilities, as our interview data show. In line with another study from the COVID-19 pandemic, in which family supportive supervisor behavior was associated with less work-family conflict [62], understanding and support by managers was described as helpful for employees with stay-at-home children.

Several aspects (e.g., business trips, digital media use) reported by the interviewees were described as both demanding, but also relieving. Although this difference might be due to managerial responsibility and varying intensities of business trips, this finding could also be explained by drawing on the idea of distinguishing between challenging and hindering job demands as an extension to the underlying JD-R model [41, 42], based on an individual’s appraisal of perceived demands [43, 63]. Similarly, digital communication (to such high extent) was characterized as a double-edged sword both facilitating collaboration and mitigating isolation but also overloading participants. Regarding the appraisal of digital media use, recent research challenges the validity of the media richness theory, stating that richer media in additional cues (such as audio-visual media like video calls instead of audio-only media such as phone calls) improve communication [39, 64]. Instead, the phenomenon of “Zoom fatigue”, i.e., perceived exhaustion from the extensive use of video conferences, has recently emerged [39]. This notion is supported by several findings of our interview study. First, employees reported that using video conferences not only for work-related but also for leisure activities and private meetings with friends and family during the lockdown exhausted them. In line with previous research [56, 65, 66], our findings also support the observation that the number of meetings has increased during the COVID-19 pandemic. Some interviewees reported that they deemed their participation in many meetings unnecessary or that meetings could have been organized more efficiently. Moreover, managers perceived difficulties in virtual leadership because video options were either not used or did not fully replace face-to-face communication. Considering these findings, media richness theory, as developed in the 1980’s, might be outdated due to today’s intense use of digital media. More cues could be overloading rather than helpful. The challenge will be to identify a sweet spot where the benefits of using it take effect but are not yet perceived as overloading [39]. As our results indicate, it should rather be carefully questioned which kind of media is adequate for which purpose (see also [67]), who really needs (and does not need) to participate to prevent perceived overload.

In our results, we observe mostly overlaps but also some differences among employees and managers. Interestingly, work content related demands were only reported by employees, not managers. By contrast, only managers acknowledged the benefit of the pandemic for their company. These results are plausible given the different focus that employees and managers have due to their roles. Similarly, individual differences about the perception of the work environment at home as either positive or negative (e.g., fewer vs. more disturbances), depend not only on the role (employee or manager) but also on contextual factors such as sharing the home office with partners or children.

### Coping with unforeseen persistent remote work

Coping strategies reported by interviewees aimed at sustaining social connections by means of digital communication media. Due to the pandemic-related restrictions affecting leisure activities, some interviewees tried out new hobbies or resumed old ones to maintain existing habits as good as possible (e.g., exercise, meditation, or gardening). Problem-focused strategies such as physical activities (indoors and outdoors) also compensated the restricted access to gyms during the lockdown. Likewise, emotion-focused coping strategies, e.g., talking to family and friends or looking at the bright side, were used to maintain a positive attitude. These insights are compatible with other findings on coping with the impacts of the COVID-19 pandemic [68, 69]. Interestingly, employees and managers only differed in problem-focused coping strategies, where managers added other strategies than those applied by employees. A possible explanation could lie in their different roles, tasks, and responsibilities they had to cope with as some of the categories indicate (e.g., finding new ways of obtaining feedback or conducting personnel interviews). While problem-focused coping differed in this study, coping strategies aiming at emotion regulation were rather independent of their job roles. As can be seen in our results, autonomy and experience can be important and facilitating resources for employees to cope with the telework situation and engage in crafting their jobs to their benefit. This is particularly interesting given the sudden change not only of the work environment but overall job demands and resources during the COVID-19 pandemic that may limit possibilities for job crafting that require organizational-level change [70].

### The facilitating and debilitating role of leadership

Regarding the role of leadership, the identified categories indicate considerable overlaps in what both employees and managers deem to be important for leadership (e.g., communication, trust, staff care). However, managers reported additional challenges such as having to invest more time for digital leadership and fulfilling a dual role, keeping performance in view, or respecting employees’ privacy. Employees, on the other hand, mentioned that they were affected by how managers’ attitude toward remote work and occupational health policies as well as the degree of autonomy granted. Overall, our findings also support the notion that self-leadership in flexible work arrangements such as remote work requires self-discipline [71]. Nevertheless, the role of managers remains important regarding employees’ health. As our findings demonstrate, some managers found it difficult to see how their employees were really doing from a distance. This ties in with findings on health-promoting employee-directed leadership (i.e., staff-care), which was found to be lower but more effective in times of crisis [72].

However, a positive relationship with the supervisor does not necessarily seem to lead to better outcomes. High leader-member exchange can even foster family-work conflict during the pandemic, especially when employees’ ability to cope was low. This might be due to managers’ persisting performance expectations, not considering their employees’ demanding work environment at home. Thus, the ability to cope also likely plays an important and buffering role regarding manager-employee relationships while working remotely [73].

### Strengths and limitations

#### Strengths of the study

The qualitative approach of our study enabled us to gain differentiated, in-depth insights into how employees and managers experienced working from home during the COVID-19 pandemic and how they coped with it. This allowed us to explore individual perceptions, coping strategies, and different perspectives on the role of leadership. Our results are not limited to the beginning of the pandemic and switching to remote work but shed light on the of long-term effects of this unexpected changeover under the conditions of the pandemic. Our predefined inclusion criteria for study participation ensured a good balance between achieving comparability and including sufficiently heterogenous perspectives. This heterogeneity is also reflected in the sociodemographic characteristics of our sample (e.g., gender and age). By drawing on both perspectives of employees and managers, we acknowledge and reveal differences and commonalities in their perceptions and behaviors. Thereby, we were able to draw comprehensive conclusions. We based our study (i.e., the interview guide and coding scheme) on a well-established theoretical framework [41–43]. Therefore, our findings tie in well with further research in this area and allow for quantitative validation. Reproducibility was ensured by involving different members of the research team in the development of the interview guide and coding scheme (see [74]).

#### Limitations of the study

One undeniable limitation of our study is the very specific pandemic context in which it was conducted. The COVID-19 pandemic has long exceeded its peak of severity (at least regarding work from home policies). One might thus argue that the results of our study from this specific context could be outdated by now. While we acknowledge this incontestable limitation, we contend that the pandemic context can be understood as a burning lens through which certain circumstances of remote work from home may become more apparent. This helps us to identify potential stressors and resources, which still have important implications for designing sustainably healthy post-pandemic remote work. These insights may also help employees and managers in potential future ad hoc crises that require flexible and fast adjustment. While the results put forth criteria for making informed decisions about when and where to employ which type of remote work model, they can only be interpreted with careful consideration of the specific context from which they originate.

Given our explorative qualitative approach, our study does not claim to be representative and might be subject to some biases. Most interviewees did not have children to provide additional care for while working from home. This distribution in the sample could be due to a selective bias, since employees may be more likely to participate in an interview study if they have sufficient (time) resources. Although some interviewees indicated they did not want to continue working from home, the majority were in favor. Again, this could be due to selection bias, as employees may be more motivated to participate in a study if they want to support continued remote work. On the other hand, employees with strong negative attitudes may see their interview participation as an opportunity to make their opinions heard. Another limitation regarding the procedure of the study might be that most interviews were conducted by telephone, therefore, providing no visual cues to the interviewer. Thus, some interviews were conducted via video call at the express request of individual participants. Another limitation that needs to be considered when interpreting the results is potential hindsight bias. Although we explicitly asked our participants to differentiate between different phases of working from home during the pandemic, hindsight bias distorting the overall picture cannot be ruled out. We actively tried to address these limitations by steering the recruiting towards such underrepresented perspectives in our sample. Despite these limitations, our in-depth findings contribute (a) by complementing prior studies following a quantitative approach and (b) by combining employee and managerial perspectives.

### Theoretical and practical implications

#### Theoretical implications

Our findings on different perceptions of several changes due to pandemic-related work from home (e.g., perceiving lack of business trips as a relieve versus missing them) emphasize the importance of considering appraisal in changing work environments. While the JD-R model only allows for categorizing findings as job demands or resources, the challenge-hindrance stressor model adds the differentiation between challenge and hindrance demands to the framework [63, 75]. However, this framework has been criticized for its a priori categorization of stressors and without considering individual appraisal and for not differentiating between hindrance and threat appraisal [76] as suggested by the underlying transactional stress theory [43] and empirical findings [77]. Yet, it seems unclear whether this distinction is empirically worthwhile [78]. Further research job demands, resources, and coping strategies in remote work could thus benefit from looking at it through a different theoretical lens and investigating individual appraisal of stressors more thoroughly. Another avenue for future research could be to look at how employees and managers deal with these demands and resources through the lens of job crafting. Such research would extend our findings regarding problem-oriented coping by focusing on how employees and managers proactively increase their person-environment fit and their well-being [70, 79]. Given the sudden change and heterogeneous picture of challenging and hindering job demands as well as job resources during work from home in the pandemic context as indicated by our interviewees, more research would be required to better understand job crafting under such specific circumstances.

Tying in with similar research, our study shows that even though employees and managers were in the same storm, they were not necessarily in the same boat [80]. With the unexpected shift to mandatory work from home, our results show that employees and managers showed high degrees of creativity, pragmatism, and experimentation to cope with the changed job demands (e.g., work environment, communication, collaboration) and job resources (e.g., leadership, access to social support, team cohesion). Consequently, personal resources may have gained in importance as employees were mainly responsible for their immediate working conditions at home (e.g., creating a healthy work environment, boundary management). Our findings indicate which resources have helped employees and managers in different ways and which kind of coping strategies were applied. Yet, future research needs to shed more light on the overarching role of coping strategies, personal and job resources in post-pandemic as well as crisis-induced remote work settings.

#### Practical implications

Given the exceptional and long-lasting circumstances of working from home during the COVID-19 pandemic, several lessons learned can be deduced from our findings that provide valuable practical implications for profound change processes, future crises, and post-COVID-19 remote or hybrid work. After three years of the onset of the COVID-19 pandemic, managers and employees should reflect on their experiences of working from home and elicit best practices for present and future work. Overall, we can conclude that technical issues have decreased over time. However, having easy access to technical support remains important. Moreover, regarding the fast-evolving nature of technologies, the sufficiency of technical equipment at home should be reviewed regularly. New technologies (i.e., hard- and software) require ongoing training for efficient and healthy use.

Regarding the biggest issue mentioned in our study, communication and cooperation, managers as well as employees could evaluate the necessity of meetings more carefully, and critically question whether a meeting is the right medium, who really needs to attend it, and who could be informed otherwise. Moreover, (online) meetings should be organized more efficiently. However, this does not mean to ignore social aspects of meetings like small talk. Check-ins at the beginning of meetings can contribute to a feeling of cohesiveness and help to make online meetings more comfortable [81]. As can be directly deduced from our interviews, important meetings and social gatherings should take place in person. This especially refers to kick-off meetings, personnel interviews, and team building events [82].

Regarding detachment from work at home and leadership through digital media, employees and managers should be supported in developing such skills. While employees need to build self-leadership skills, managers need to be aware of their role model function, especially regarding health-related behavior [83]. This includes establishing communication rules in the team and adhering to them [84] as well as considering individual segmentation preferences [61, 85]. Nevertheless, and precisely because of their role model function, self-care also plays a vital role for managers. As leader self-care is associated with higher staff-care (thus, better health and lower strain among employees), staff-care should not be pursued at the expense of managers’ self-care [86]. Accordingly, managers themselves need to be trained to improve and maintain self-care.

## Conclusions

The outbreak and persistence of the COVID-19 pandemic have changed the world of work profoundly. The unforeseen consequences generated by this exceptional situation have placed unique demands on both employees and managers. In this unprecedented new work situation, many have found creative ways over time to adjust and have come to appreciate advantages of working from home. Our study reveals how employees and managers leveraged new resources and developed new coping strategies in face of adversity. Given the extreme boundary conditions of the pandemic, lessons learned deduced from the study results provide guidance for coping with profound change processes and the design of health-promoting post-pandemic work, striving to balance the benefits of both office and remote work.

### Supplementary Information

**Additional file 1.** Overview of Identified Demands, Resources, Coping Strategies, and Role of Leadership.

## Data Availability

The datasets analyzed during the current study are not publicly available due to German national data protection regulations but are available from the corresponding author on reasonable request.
