# Supplementary material for: “The magic triangle between bed, office, couch”: a qualitative exploration of job demands, resources, coping, and the role of leadership in remote work during the COVID-19 pandemic
Source: BMC Public Health. 2024 Feb 15;24:476. doi: 10.1186/s12889-024-17995-z (PMC10868004; doi:10.1186/s12889-024-17995-z)
Supplement: Supplementary file 1 — Additional file 1. Overview of Identified Demands, Resources, Coping Strategies, and Role of Leadership. [file 12889_2024_17995_MOESM1_ESM.pdf]

Supplementary Information – Additional file 1: Overview of Identified Demands, Resources, Coping Strategies, and Role of Leadership

Table 1. Overview of Demands

Category	Demands	Interview Quotes
Work Content	Deployment planning	"[…] the scheduling of colleagues, i.e., who has to be ready on the phone, when, and how, and of course the colleagues first had to be equipped with the appropriate technology for home. That was a challenge. How do you schedule the colleagues who can already make phone calls from home? Or are still in the office." (employee #6, female)
	Conflicts with customers	"Yes, what was still a bit different in the office, in the office you could come and knock and say: 'Here, I have this and this problem'. [...] So the bottom line is I have to make a decision for my team leader. Or I leave it here, but some things we can't leave here because we are in the disruption periods or customers who escalate, and we have to make a decision. [...] The problem or the escalation or, if the customer is yelling and fussing around here, there's everything." (employee #15, male)
	Lack of business trips	"Exactly, my customer, for example, is based in the UK. So, I was used to at least having video calls with them, so nothing has changed at all. And otherwise, so what I just miss is seeing my colleagues at work and we just used to have team events and I was also at the customer's a couple of times a year and stuff like that is of course missing, but how the company is supposed to improve that now, yeah, that's difficult." (employee #7, female)
Work Organization	Loneliness in home office	"The only thing that was a bit unusual at the beginning was that everything is pretty quiet here, maybe the radio, but we also had it on at work. It's just that our office is a bit bigger, and we're sitting with a few people. Of course, everything is much more restless, because someone is always on the phone or talking to someone else. And here sometimes, you think, oops, you don't hear anything ((laughs)). That's actually what was the most like that. And that you sometimes look and think: Oops, how are you actually walking around here? But nobody sees ((laughs))." (employee #13, male)

	Collaborating digitally with clients	"So actually, changing the organization so that you really work 100% digitally. Also, organization, coordination with colleagues. Otherwise, you would quickly meet somewhere, discuss something, and now you had to somehow manage it by phone. Even with the clients, it was sometimes a bit difficult to reach them, because they themselves had difficulties due to the pandemic." (manager #12, female)
	Additional digital exchange formats	"I still found it [digital exchange formats] stressful to some extent. And it didn't have the same relaxing and loosening-up factor as the real conversation at the coffee machine because you're not away from the medium, and as soon as I sat at the computer and then perhaps had stressful phases from the projects, I felt it more as an additional burden. And I thought, 'Wow, here comes the next coffee call again, but I actually have way too much to do.' And I think you very quickly got into this whirlpool of, 'Ah, I don't have time for this, I just have to work through this, work through this,' and I think you sometimes really forgot to take care of yourself." (employee #2, female)
	Digitally instructing employees	"[…] I found instructing colleagues very, very exhausting, because it's much easier to explain a work step or something to someone if you just sit next to them and do it once with the person, instead of somehow trying to explain it over the cell phone, because especially at the beginning it was so that we didn't have the possibilities to share the screen with teams or Skype or something. It was real, we each had a cell phone, then called each other and then without seeing what the other was doing somehow explain work papers work steps I found very, very exhausting and very, very difficult." (manager # 13, male)
	Digital communication is unfamiliar	"So actually, I found that the worst, I'll say now, I was actually sitting in the office with four other people and, if I had a problem or a quick question, I could very quickly ask across the table, 'How's this? What does this look like? And what do I have to do?' And then all of a sudden you were sitting here by yourself and, if you wanted to ask someone, then you always had to call them and that's when that overcoming was just a little bit bigger then, to say, okay, are you really calling someone now for this, for this little question that you just have?" (employee #10, female)

	Digital communication is not able to replace face-to-face interaction	"That personal, those nuances that are in between that just can't be conveyed at all when you're over the phone or even when you see each other like that now, that's just so much more that you get when you're sitting together." (employee #11, female)
	Digital communication formalizes interaction	"So, by not just being able to walk in and have a quick chat and everything was kind of always on, 'Is the light on teams red or green now? Is it available or not?' these many ad hoc conversations, topics and briefings have actually disappeared, and it has become a bit more organized. Sometimes too organized, so as I said, it's all become very formal, it can't be as agile and fast, but there's also not as much ad hoc stuff coming to a table. People think twice, even the boss: 'Do I send this in now? Do I call them? Do I write them an e-mail?' It's all more complicated than just coming over for a minute." (manager #3, male)
	Lack of professional exchange	"Otherwise, you'd hear from your colleagues over their desks what topics they were working on, what challenges they were facing, what was going well, what was worth mentioning? And of course, that was missing a bit, this simple chatting across the desk." (employee #6, female)
	Complying with working hours and breaks	"So, the thing that is more difficult for me, and I think it's the same for a lot of people, is somehow organizing your working hours better. That is, that you don't work too much or too little. Because for me it is often the case that I have a time where I - somehow a colleague is on vacation for three weeks and I actually work for two people. And then my contact person from the customer is on vacation and then I can also relax. And in the past, because I left work after eight hours or always tried to, I had a pretty good overview. And now, in fact, since I'm also at home, it all kind of gets lost." (employee #7, female)
	Not getting to know new colleagues	"Actually, we have a relatively good team dynamic in our department, but we have also gained a lot of new colleagues, for example, who I don't really know yet, because you just run into each other in the office and then you start talking and get to know each other a bit. And when you're sitting at home, it happens less often that you think: Oh, I'll just call the new colleague." (employee #10, female)

	High frequency of (video) calls	"Now, of course, this [remote communication] has become more intense in the home office, and what is particularly intense, of course, is this close succession. I think it's the same for many people, regardless of whether they're managers or employees, that you have a lot of conference calls from early in the morning until late at night and hardly any breaks, or sometimes I make another phone call at the same time." (manager #7, male)
	Commute time missing	"Well, I haven't been on the phone the whole time [when commuting], but the phone calls I have to organize rather laboriously ((laughs)), in quotation marks, in the home office. Then I don't get on it quite as quickly […]." (manager #7, male)
	Impeded vocational training	"And I can't send trainees to work from home. And that is very difficult. So now I have a trainee who sits all alone in the office because the whole team is working from home. […] I can do that because I know he's reliable, and he'll ask if he does not have any tasks. But that's an individual decision. If this were to happen across the board with other trainees, then we would no longer be able to do our training in the way that is simply intended by law." (employee #1, female)
	Impeded information flow	"So, we sit here [in the office] in one room on purpose. The communication is extremely high. And, if one person is on the phone, the rest of the people hear that and then we're just like, 'Where was that now?' 'Yeah, that was there and there.' 'Ah, all right.' And then you're informed. That's all eliminated now." (manager #2, male)
Social Relationships	Being new (onboarding)	"[…] I haven't been able to get to know my colleagues very well yet. I talk a lot on the phone with one colleague, the one with whom I work most closely. I simply have to coordinate with her a lot. I think that's why I've gotten to know her a bit more personally in the meantime. I only have e-mail contact with everyone else. Even across departments. I know names from e-mails, but I don't have a face to go with them." (employee #18, female)
	Lack of support from supervisor	"The boss was more like, 'Yeah, well, that's [implementing remote work] what you're here for now.' Before that, he also treated it very neglectfully. In other words, the topic of human resources in general. He's a salesman, we're a sales company. And he's pretty good at it. And HR was just a bit of a sideline, so no, there was no one who had even the slightest experience with such things." (manager #1, female)

	Lack of contact and private exchange among colleagues	"[…] simply the communication with the colleagues has suffered altogether very much, because simply this whole times five minutes to stand together at the coffee machine and also outside of work times a tick to chat, that is just completely broken away, but if one has spoken then times with a colleague then it was actually always to technical questions then, also as soon as the technical was discussed one has hung up again. Which is why it then became, yes very, very limited and very lonely, too." (manager # 13, male)
	Deteriorating team spirit	"So, the will to turn on a camera, an order is doubtful from my point of view. Is not necessarily purposeful. I have felt a clear deterioration of the team structure, clearly. So, the team cohesion was very strong before. Due to the organizational change, the team has of course been reorganized, some have joined, some have left, that was of course an aspect. But overall, the team structure has deteriorated considerably. And we were able to hide a little further away." (manager #8, male)
	Little contact with supervisors	"So [keeping in touch] has actually always worked well with the colleagues themselves, but with the team management I have to say that feedback is always a bit lacking. So, the feedback and the fact that one reports back. Because there are many things that you can do when you make a morning tour as a team leader or as a boss, as you would like to call it, you can absorb all the frustration of the colleagues and say: 'Man, you have the problem, you have the problem'. And that makes you feel different in the end, you feel a little bit better then." (employee #15, male)
	Conflicts among colleagues	"In our case, it was also the case that some went to work from home and some stayed on site, and there was also some real resentment. So, it was like this: 'Why is he allowed to stay for months now?' And, 'I want to have a go, too.'" (employee #1, female)
Work Environment	Lacking procurement of work equipment	"What was challenging was that some people, well we warned in time, there is a shortage of electronics simply. They can't get computers, they can't get notebooks right now. So now by now it's going again, but the last four, five months have been stressful. The one you got was 300 euros more expensive than normal and that was the last scrap. So, the reviews were also like, 'Yeah that's called recycling.' ((laughs)) You get a really fancy device, the screen is beneath you, way too dark. The webcam is eight years old, they weren't even in cell phones back then, it's that bad, and the processor is up to date. So, and then you're like, 'How does this fit together? What's that all about?' Cameras weren't even available in the third quarter and fourth quarter of last year.

		It started at 600 euros in some cases, for a camera! And we fortunately reacted in time, but that was also a bit of a gamble." (manager #2, male)
	No access to work materials at home	"The challenge is, of course, that signatures, especially for annual financial statements, are still signed in the original and if there was something, then you actually have to go to the company and sign on site. Those were the days when I was there on the weekend ((laughs))." (manager #15, female)
	Insufficient work equipment at home	"At home, of course, you simply don't have the same ergonomic options or equipment as in the office. In the office, I stand up a lot or walk around a bit when I'm on the phone or talking about something. Here in the home office, I have my normal desk, which has only one height and I sit here on my chair, which is also good, but it is something else. And ergonomically, the light should somehow come from the side. It's not like that with me. You see it shining from behind. It's just exhausting." (manager #10, male)
	Not being alone working from home	"That's why in this case and by the fact that I still had the influence children on site here, it was a very unpleasant experience for me. On the one hand, that has to do with concentration, focused work, and on the other hand, that actually has to do with the leadership activity, because I felt uncomfortable and not good with leadership at a distance." (manager #8, female)
New Forms of Work	Lack of experience with working from home	"Yes, so data protection compliance, then I have to comply with the workplace ordinance? How do we do that with the equipment? Does everyone even have enough data line? How do they go in? Do they go in with Wi-Fi or with a cable? Do we even have enough cable? What agreements do I have to make? At that time, there were no internal documents. So, the very first thing I did was to introduce them and then I had to start with something special. Then, who has circumstances of a personal nature? Who can do it? Who wants to? How do I record the working time if it's not like that? Yeah, that's quite a bit." (manager #1, female)
	Digital leadership	"So, as I said, basically much more time spent and still, so for the individual employee, so still much further away from the individual employee. The closeness has been lost. There is no longer any direct leadership, in terms of human leadership, which perhaps also affects a bit of duty of care, because I'm also worried that one or the other person in the team might slip in one direction or the other." (manager #8, male)

	Having a bad conscience working from home	"[…] you watch yourself and say, 'Gee, now I've been chatting with him for so long again,' and in the home office you also feel a certain obligation not to go overboard, so that you don't make a bad impression working from home. At least that's how I feel. And if you then talk to someone for a very long time, then it is already, then you also get somehow such a bad conscience, I must say. Then at some point you say, 'Okay, you, I have to move on.' But if you transfer it to your work, what was it like in the past? Then you sometimes talked just as long, but then the boss probably even joined in and chatted for a while and then you didn't have this, you didn't have this bad feeling about it, you have to say quite clearly." (employee #12, male)
	Lack of spatial and temporal separation	"[…] I noticed that it's extremely difficult for me to go directly from 'I've now worked' to 'I'm now doing my leisure stuff'. Because normally I have such a long break in between, because then I leave the office, I'm in a completely new environment, then I go home and by the time I get home I'm completely set up for leisure time. And now it's just really like that, I turn off the computer and could theoretically take two steps to my bed and then the free time would start there." (employee #10, female)
	Work in office more difficult	"I actually manage everything a little fluffier and better in the office than I do in the home office." (employee #1, female)
Further Demands	Pandemic-related demands	"And then especially in the lockdown phase, when you try to go out as little as possible anyway, especially in winter, when it was already dark so early and you don't really feel like moving around and you're still sitting in your home office all day and maybe just walk from the bathroom to the table and then back to the fridge. Yes, that's when I noticed that it also puts a strain on me psychologically, when you simply don't experience as much anymore and so on." (employee #7, female)
	Uncertainty about duration of pandemic	"[…] because you didn't know what was coming, how dangerous the whole thing was anyway, so from a health perspective you were just actually grateful that you didn't have to go [to the office]." (manager #15, female)
	Lack of clarity about remote work regulations	"[…] we [the company] never talk about telework, but always about mobile working, because then of course nothing has to be sponsored. So, these are issues that came up in our company, where I asked myself: 'Why do I have to invest in a more ergonomic mouse now? Why do I have to think about buying a more comfortable chair? Wouldn't that actually be an employer thing?' But yes,

		you're not obligated to do that when mobile working, so 'off the hook' I think at this point ((grins))." (employee #2, female)
	Office work less productive	"But even now I have to say that I was only in the office last week and when I compare that, I get a lot less done. Simply because you're excited to see your colleagues again, you're chatting away again. So, I think this focus has simply shifted. What was perhaps normal in the past, standing by the coffee machine and having a chat here and there, is simply no longer something you're used to. At home, you just work all the time, you might not have that exchange either, and now you perceive that as being unproductive when you're in the office, actually." (employee #2, female)
	Increased (private) digital media use	"Well, I'd say that for the private sphere, life as a whole has been completely relocated to telcos. I do a bit of volunteer firefighting on the side, and at the end of the day, if you look at it that way, we've also switched everything over to telcos in the private sector. I'll just call the member entertainment or the services we did on telcos at that moment. It's just like it was in working life, that you mainly worked with telephones here." (employee #15, male)
	Need to cook	"We have always had a canteen at the location in (named location). And that was of course really good. And now you have to take over this canteen function yourself. And if you don't just want to eat fast food, I think it's relatively time-consuming, even the planning, the preparation. I then really look: 'Do you really have so much time at lunchtime to cook something again or do you somehow make it ready the day before and then microwave it?' Because I don't want to eat fast food. Or I make two salads in the morning and put them in the fridge so that I can take them out myself. So, I think that's a logistically higher effort for me. Because I also make sure that I eat a reasonably healthy diet." (manager #9, female)

Note. Categories presented in italics were mentioned by managers only.

Table 2. Overview of Resources

Category	Resources	Interview Quotes
Work Content	Participation in the change process	"So, I even have a different view than just: I've been transitioned, and I've been working from home. I was also involved in making it possible for all people in our company to work from home. Because our IT department made sure that within a week, and we received a lot of praise for this, all of our employees were able to work from home. And the changeover was surprisingly relatively harmless for me […]" (employee #4, male)
	Enjoying one's job	"I enjoy it and I actually enjoy this dispatching and this moving people back and forth, orders and organizing a little bit. That's my world in that direction. Sending someone there or getting material there or everything that comes in that way. So, I don't have a point where I have to motivate myself strongly. I see it as my daily business and I enjoy it at that moment." (employee #15, male)
	No more business trips	"That we then just, we can't just meet now in one place, some people are hours away. We've also realized that ourselves. And I've come to love that, by the way, in part, that I don't have to travel so far anymore." (manager #5, male)
	Benefits from pandemic	"We were lucky because we were in an area that was not negatively affected by the pandemic. For us it was rather the opposite, we now have twice as much to do, because we not only maintain the (incomp.), but also the home offices of the employees in addition. And we had a lot to do before and you could also see that, so money alone doesn't make you happy ((laughs)). So, we paid out a bonus." (manager #2, male)
Work Organization	Quick implementation of remote work	"[…] it was decided early on how to deal with it and what all the prerequisites and effects it would have on our further work and activities. And from there one is ultimately, because it was all such an uncertain time, so we were bombastically well prepared, because we all already in the run-up, I think the year and the one before we switched our rollout from all the stationary computers to laptops, so that was not a big effort for us to collect there completely immediately fast everything and to work from home." (manager #10, male)

	Fewer disturbances at home	"Because I'm a person who is very easily distracted, which means that with the move to a larger office and correspondingly more colleagues, the noise level has naturally also increased. And that's where I had a lot of problems. This move to the large office was shortly before the start of the pandemic, so to speak. And I had my stomachache with it because I kept working with headphones, simply because I couldn't concentrate on my work. And accordingly, that was almost like a relief for me to then be able to sit down at home in a quiet environment and really work super focused." (employee #2, female)
	Breaks are more relaxing at home	"Another aspect that I noticed is that before, during the breaks, i.e., half an hour, hour lunch break, that was always work for me, because I used this time to make short coordination, to somehow take new colleagues with me and then to eat together. And I didn't really have a break where I could really switch off, but I was always working through the day. Because as a human resources manager, it's always about cultivating relationships. And I don't have that in the home office, where I can simply turn off my cell phone for half an hour and I'm not reachable. And then no one can intercept me in the hallway, or I think, 'Oh, cool, now I can reach the Board of Management, where it is right now, then screw the lunch break, but then I can clarify something. So, I'm more conscious of taking breaks again now." (employee #8, female)
	Working time recording system	"So, we have a clocking in system, so we clock out. I clock in early and clock out at lunch and clock in again after lunch and, when I clock out, I turn off the computer." (employee #4, male)
	Digital communication	"On the other hand, the fact that I am responsible for two regions means that I am, and I don't mind admitting it, sometimes in conference calls or phone calls at the same time. That's an advantage of home office, of Corona, because when I'm present somewhere, I don't go to another conference call, well then you leave sometimes. But then I can use that a bit more in these corona times. Of course, that's not always so ideal, but well, I weigh it up and the chances outweigh then now and then to be somewhere at the same time. Because I don't have to move spatially, I can do everything from my desk." (manager #7, male)
Social Relationships	Support from colleagues	"I would say that my colleagues made it very easy for me. There was a great deal of understanding when things didn't work out at the moment, and also that you knew you simply had to take time for certain things that otherwise just happened on the side." (employee #17, female)

	More equality among team members	"On the one hand, with the team in Germany, where I also had very physical and close contact before, you automatically distanced yourself a bit. But now, when we have a big team meeting across the markets - before it was just the German team sitting in an office and the rest of the teams somehow joined in via [Microsoft] Teams - suddenly everyone was the same. So, no one was close to the boss, I would say, because everyone worked virtually, and that led to a bit of a convergence, at least between the teams in Austria, Poland and Switzerland. To me and to the whole team structure." (manager #3, male)
	Support from supervisor	"[…] it was all work all the time, the whole day, and that in the private sphere. And that really got to me, and then I also looked with her [the supervisor] help: Okay, which topics are so emotionally stressful right now, which ones can we perhaps pause or pass on or perhaps also downgrade in the prioritization, so that it is no longer so intense and thus less stressful. So that's where she supported me. Yes." (employee #8, female)
	Seeing colleagues in office when possible	"Well, I've started going back to the office now and then, because we're already allowed to go to the office now, so we have a planning software now where you can reserve a place, then it's practically blocked for you for the whole day. I don't go every week, but I do go every few weeks now, just to see some faces in real life. So, I already notice that I miss a few colleagues. Now I just have to do something with them during the break, because it used to be such a habit. You go out with the colleagues with whom you get along well, you go out for lunch or do something else during the lunch break." (employee #5, male)
	Regular contact with supervisor	"Um, well, I thought my direct supervisor did an excellent job. Because he also, he just, we had an appointment every two weeks where we just talked to him about how things were going for us at the moment and whether everything was still okay, whether anything was bothering us. And in my case, that often turned into just talking about God and the world for half an hour and just exchanging ideas. So, it's kind of a little substitute for what you would have otherwise had at work." (employee #3, female)
	Strong team cohesion	"And I feel like the pandemic bonded us together as a team because we really overcame a lot of depths and a lot of challenges." (employee #8, female)

	(Digital) team events	"For example, last year in December, I said, 'Here, come on, Christmas party we're not allowed to have, and don't we want to have a virtual Christmas party like that here?' And half the team thought that was dumb and the other half said, 'Come on, let's do it.' We said virtual mulled wine drinking, that's it. And I really have to say, that was really funny. Then I got a card game with questions where you had to guess something, and I think we spent three and a half hours there together. So, it was really funny. It was a super experience, I must say." (manager #9, female)
	Trust	"So, I just thought it was cool of her [the supervisor] that she trusted us 100%, that we can decide ourselves how we work and from where we work. She didn't tell us to come to the office, she didn't tell us when to work, there was only the appointment once a day with the daily. Other than that, she trusted us completely. And that trust motivated me a lot." (employee #8, female)
Work Environment	Provision of work equipment at home by employer	"Yes, we got a laptop from our employer that we can take home with us, where all our programs and so on are on it. And a headset that we can connect to the laptop, and I got a screen, a big one. So, we would have everything we need for that. I already had a mouse or something from home, but we could have taken everything with us." (employee #16, female)
	Availability of IT support	"Yes, well, that actually went relatively quickly. On the first day, we actually got the most important things fixed so that we could work. We simply got in touch with IT and they were actually able to help us as far as they could." (employee #16, female)
	Importance of being well-equipped at home	"So, I'm personally relatively well equipped, I'm also an IT-savvy person, so I have the appropriate equipment at home. What I decided to do, I still had two normal 19-inch screens at home before the home office and was able to get a good bargain, so now I've also treated myself to at least a 24-inch screen." (manager #6, male)
	Having better equipment at home than in the office	"Otherwise, I honestly can't say, because the general conditions have improved. So, with monitor and keyboard and et cetera." (employee #1, female)
	Financial and material support for work equipment by employer	"And from our company, but they sponsor a part to such an office swivel chair, at the company (names company) you get it cheaper and then I bought it myself." (manager #9, female)

	Digital collaboration tools	"So, the thing that's really very positive, we just introduced Microsoft Teams last year, I think it was early last year. And that's just a great function to kind of chat with people and also to have an overview of everything. And, if it hadn't been there just before Corona, then I think everything would have been much more difficult and so you can also create digital appointments relatively easily and have different chats with different groups. And in the past, we had an outdated tool where messages were archived relatively quickly, and you couldn't use the search function. So that's definitely been very helpful, a good digital tool to stay connected." (employee #7, female)
	Digital documents for external collaboration	"But well, fortunately, we have found that something has changed at (names contractor) in the meantime, apparently also pandemic-driven. Because before that was also, as I said, a multiple printout that we had to send over, but now you can also do it in digital form. So, others have learned as well. Now I'm curious to see whether (names other contractual partners) also gets it right and then ((laughs)) maybe even something will happen." (manager #6, male)
	Workspace directly available at home	"No exactly, the setup times, that's what I was getting at, we have these setup times, this desk sharing. That means we have to set up our laptop every day at the workstation, connect it to the monitors there, with a separate keyboard and so on. For hygienic reasons. And that also takes up a bit of time. In the worst case, you have to get a small trolley out of a small safe, where your laptop and everything you need is in it, then you look for the workstation, which you have to book in advance, then you work there, of course you have to set everything up beforehand, wire everything, connect the monitors to the docking station, the mouse, keyboard, webcam if necessary, the headset and you haven't seen it. And then you work and then it's the same thing backwards again. That's just such a little thing, of course, but that's an incredibly noticeable process because that just sucks. It's really just a total little thing and there are really worse things, but it's one thing that really gets on a lot of colleagues' nerves because it's also so ineffective." (employee #12, male)
	Opportunity to work standing up at home	"Nothing has changed from sitting to standing [compared to the office]. I can also stand a lot during conference calls and the like. It all fits." (manager #8, male)
	Benefitting from experience with digital work	"So, our department is represented across three locations […]. So, we've always done a lot of things via videoconferencing and telephones and so on. We rarely ever sat down together in the office, in a meeting room or something, and discussed things. So, the change, in terms of

New Forms of Work		videoconferencing and stuff like that, wasn't so much or the challenge to exchange about it." (employee #6, female)
	Freedom in self-organization	"And yes, I actually find it a bit more relaxed to work at home, because you are not so controlled by everyone. So of course, there are two sides to it, you can't ask questions so quickly or anything, but I find it a bit more relaxed in general, because here, let's say, you work on something light and then listen to quiet music, which might otherwise disturb others in the office." (employee #16, female)
	Saving time	"In addition, then there are the commute times, I had already said, and another advantage is, when you finish work at some point, you finish work, then you also finish work immediately, you then simply go down from the attic in my case, are at home, can take care of the children or the like." (employee #12, male)
	Getting used to working from home	"I would say you get used to it, to the situation. So, a very strong routine has happened. For example, I was in the office yesterday and it was totally unfamiliar. To get up early in the morning, to get ready, to think of X things, to have the way to work and so on. So, somehow, man is a creature of habit and the habit of going to the office every day has shifted to: 'Okay, home is the center of my life. And everything really only takes place at home. That's why I would say, yes, that has changed completely. So, all the routines that still go hand in hand with each other." (employee #17, female)
	More spatial flexibility	"We have multiple locations now because of the merger, even going to (named other location) and working out of the office from there for a week and then kind of putting your feet in the water after hours on the side [is possible now]." (employee #8, female)
	More temporal flexibility	"And if an e-mail comes in at 5 p.m., I might still read it, but in contrast to a year ago, for example, I don't think about it so much after work because I'm simply better able to categorize it. [...] But I realize that this way I find a good mode between: Hey, I'll take a break for an hour at lunchtime and then check in again in the evening or answer quickly. Or, if I have a creative phase on a Sunday afternoon, because it's just quiet and I come up with a few ideas for the concept, then I somehow briefly do that again on a Sunday evening. And then I take Thursday afternoon off for that. So that's super flexible. And before, I was a bit afraid that it wouldn't work. But we have a corporate culture where it's really about doing the work. And when, how and where you work is up to you." (employee #8, female)

	Integration of work and private life	"I can take the kids to kindergarten without having to rush off, I'm back with the family sooner, I can take care of appointments sometimes. And in general, I can better combine the private with the professional." (employee #14, male)
	Being able to separate work and private life	"So, I'm a friend of separating my professional and private life and not firing up the computer again at ten in the evening to see what's been going on, but when it's the end of the day, that's the end of the day." (manager #15, female)
	Saving money	"You simply have less stress due to the travel time and so on and so forth, you don't move your car as often, of course, and therefore have lower fuel costs, those are just the classics." (employee #12, male)
Further Resources	Family	"For me, that was, I missed that whole social aspect. So, I was quite happy to have my husband at home and also actually the cats." (employee #3, female)
	Workplace health promotion offers	"We had online counselling on how to set up the workplace to make it more ergonomic. Then we have an anonymous offer from an institution that employs psychologists and psychotherapists, so that we can seek help there at any time if something is wrong. Also, with our partners or similar, which I didn't take advantage of myself, but which I found very good. Especially because I had the impression that my company is really only interested in its employees being well and not that they, I don't know, want to find out who is about to collapse ((grins))." (employee #3, female)
	Children's sick days for parents	"We had a certain time when the kindergartens were only open to a very limited extent or not at all. We solved this, for example, through the child sick days that existed, where I was not on duty, or my partner was. What we didn't do, for example, which was also propagated, was to look after the children at home at the same time as working. Experience shows that it doesn't work, at least not for me, that I can work productively with two small children at the same time. (employee #14, male)
	Trainings on digital leadership	"I took part in a seminar that is already starting to be held here: 'Leading hybrid teams'. So, office staff, field staff, especially now at Corona times, how you can simply recognize certain things, also as a manager. Are the employees doing well? Are they not doing so well?" (manager #5, male)

	Pet care	"Well, I make sure that as far as tasks can be completed, they are completed. And I also finish them on time. But I also have some personal obligations because of my dog, who shoos me out and says: 'I'd like to finish now, please.' ((laughs))" (manager #12, female)
Personal Resources	Experiencing meaningfulness in job	"I thought the feedback was totally nice from them. At first it was a bit of skepticism [on the part of colleagues], 'Oh God, HR is calling. Is anything happening? We have Corona, we might have to go on short-time, I don't know.' And then to hear [from me], 'Hey, I just wanted to hear how you were doing.' That triggered such an incredible, 'Yo, that's totally cool that you guys are doing this,' and to kind of feel that gratitude in that moment, then it re-motivated me for this meaningfulness, this job that I'm doing." (employee #8, female)
	Grateful for protection measures	"And I experienced that as totally positive, because it's also a kind of protection towards the employees as well, that you're at home in a safe refuge, so to speak, and don't have to face the daily office routine anymore." (manager #11, female)
	Self-reflection	"Well, maybe what I did a little better is that I dealt with myself a little better, because I just spent so much time with myself. Even though you work at the same time, of course, but it's always something else when you work unobserved than when you always have people around you. So, I think you just reflect on yourself a bit more, which I actually think is quite good." (employee #10, female)
	Serenity	"I think I coped relatively well with it because I can accept things and know that it's okay and that's fine for me. My partner was there, she caught it even more, she was also an essential driver that we simply move and need a third room. And exactly. But for me, I managed quite well. So, I missed (incomp.), of course it was a pity, but I can just accept that, find it good. Exactly." (employee #19, male)
	Mindfulness	"Or that you actually consciously look out of the window and notice a bit of what's around it, have other stimuli. I think that's actually quite nice too." (manager #12, female)
	Self-discipline	"What I often heard was: 'Then you can work in your pajamas or in your sweatpants'. So, I think you need to have a bit of structure, to really get ready, and that's what I've done, especially by going out, getting dressed and sitting at my desk in my clothes and not in my pajamas. So, I think a bit of self-discipline [is necessary]." (employee #11, female)

	Being used to sitting for long time	"Well, I used to sit at the computer a lot, but now I do even more than before, because many of the things that you used to do with people are now done on the computer. I just do other things on my computer outside of work. It doesn't bother me and it also helps that, well, I feel I see my wife more than before, so that's rather positive." (employee #4, male)
	Support in home environment	"Support in the private sphere as well. Because I am sometimes in conferences all day, I also have to be present. I have also received private support from my parents-in-law and parents in the form of childcare. Otherwise, I don't think it would have been possible to do it for so long." (manager #11, female)

Note. Categories presented in italics were mentioned by managers only.

Table 3. Overview of Coping Strategies

Category	Coping Strategies	Interview Quotes
Problem-focused	Compensatory physical exercises	"And sometimes I catch myself not sitting properly and I know that it's not healthy the way I'm sitting and sometimes I think I should pay more attention to it or maybe get another chair or maybe do some balancing exercises or something. I did that a bit at the beginning, but now it's tailed off again, I'd say." (employee #11, female)
	Introducing digital exchange formats	"We set ourselves a one-on-one once a month. We have dailies with the whole team, so always a quarter of an hour a day, so quarter to 12. We have a team meeting, which means I see my team digitally every day. And we have many coordination meetings or one-on-ones with each other. For example, once a week I have half an hour with the organizational developer where we simply exchange ideas." (employee #8, female)
	Proactively signaling availability	"It's also a lot of soft skills, interpersonal, and that has a different effect when it's in presence. But then I realized that it is possible. You just have to structure yourself very much and keep making the offer, as if: You can get in touch with me at any time, you can call me at any time, but then on the other hand you also have to have this switch, hey, I'm going to call all the employees in the company during this pandemic. So that's our goal as a team, that we really just talk to everyone, 'Hey, how are you doing right now? How are things going with your tasks, with your team? How are you doing interpersonally? Are you feeling lonely? Do you need anything? Can we support you?' And then we set up a list of crisis telephone calls, so to speak. Simply to motivate ourselves to make these contacts, to keep up with them. That was one of the first strategies to stay on the ball locally." (employee #8, female)
	Making files digitally available to all	"It also worked well that we worked our, we had a Kanban board in advance, so haptically really, there was an analogue board on the wall, that we could convert that digitally." (manager #8, male)
	Resuming pre-pandemic habits	"And after a few days I thought: 'No, who knows how long this pandemic will last and you're going completely to rack and ruin here, it doesn't work like that'. ((laughs)) And then I forced myself to take up these old habits again. This really getting completely ready in the morning and only then sitting down in front of the computer." (manager #9, female)

	Adhering to routines	"So, I really pretended to go to work every morning ((laughs)). I mean, I went to work, of course, but to my own office. But I got up at the same time and also the routine was pretty much the same. So, I also got ready in the morning normally as if I was going to the office and I didn't even start with the sweatpants and let that slide right away ((laughs)). So, from there, the process was actually the same." (employee #9, female)
	Scheduling breaks and end of work	"Yes. Well, I also had a few weeks or months where I didn't find this boundary for myself at all and, for example, with my children or we have now since the swimming pools and so on have opened again, we have a fixed appointment on Mondays, for example. We leave at half past three, I finish work and go to the swimming pool. And for me, that's when I have to schedule it so that I do it. Because if I don't have anything special planned for the afternoon, for example, then it can be good that I just continue working. And I need fixed appointments, even in my private life, where I can draw the line exactly: That's it for me, and I'll start again tomorrow morning. Just as it used to be in the office, so to speak." (manager #11, female)
	Regulating availability during leisure	"[…] I also actually started to make an appointment in Outlook for 6 p.m. or something where I then said, okay, and when it's 6 p.m. or 6.30 p.m., that when it flashes and says, hey, the appointment is coming up, that I then had a blocker, so to speak, because now you should make sure that you finish your work: 30, that if the flashes and says; hey the appointment is pending, that I had then so to speak there a blocker because now you should look that you finish work." (manager #13, male)
	Spatial boundary management tactics	"That means we also have private computers, and we also already had an office set up [at home]. That was originally a shared room, which then changed after a few months to two different rooms, because fortunately we also had a second room. Because it just wouldn't have helped the marital peace if we had continued to stay in one room ((grins))." (employee #3, female)
	Putting work equipment away after work	"[…] I have a foldaway bed and my table is also so relatively foldable. That means that on most days I really fold everything up after work, put my laptop and keyboard in my work backpack, sometimes fold up the table and then unfold my bed again, so that my room, so to speak, even if it's the same room, looks different during the day than in the evening, which I find to be quite an advantage all the time, because I somehow never have the feeling: 'Wow, I'm living here in my office or the work is coming home with me.' But rather, through such a few moves, the room is

		restructured after work in such a way that it's no longer an office, but my room." (employee #18, female)
	Upgrading work equipment	"As a result, I simply had to switch to a 50 thousand line, I think. That wasn't the case before. And I've moved away from Wi-Fi. So now I've actually laid a LAN cable and that makes sense, because Wi-Fi is unstable for something like this to be permanent. Sometimes it's okay, but permanently it was very tiring. So I turned that off." (manager #1, female)
	Creative solutions for improving work at home	"So, you sit straight for the first 30 seconds and then you slump and depending on how concentrated you are working or what kind of activities, so I don't know working with an excel sheet on a small screen is of course deadly for your back ((laughs)). When I'm on the phone, it's okay. I also make sure that I stand up. Provisionally. I simply turned a laundry basket upside down and put it on my kitchen counter and put the laptop on it. So, it all kind of works." (manager #14, female)
	Putting down headset	"But from there, in some conferences where you don't always necessarily have to say something, then I also switch to the normal speakers from the laptop to some extent. Just to clear my head and not be so shielded all the time." (manager #10, male)
	Using private material	"A few things have to be printed out or signed. Interestingly, you can also do this from home. Sometimes I have to sign a contract, then it's sent to me by post and I put it in a bag, if I'm lucky there's already a stamp on it, otherwise I just get it and walk to the post office. So, in that respect, there is no difference." (manager #7, male)
	Arranging office use	"No, there really isn't, but we simply have an Excel tracker for the team, where everyone enters when they are on vacation, when they are in the home office, so that it is ensured that there are never too many people in the office, so that you don't fall below this 30% rule. Then there is a tool where our vacation days are entered, where we also enter our home office." (employee #7, female)
	Pooling office tasks	"Well, I've also been in the office in between, of course, but mostly I've done that on weekends when no one is there, where you've exchanged documents, because not all clients are paperless yet." (manager #15, female)
	Planning tasks according to location	"So, what actually helps me is when I have a lot of day-to-day business, i.e., when you're just typing out, I'll just call it, some kind of stupid Excel lists, where you're just busy in terms of time.

		But my position is also often set up in such a way that I'm in charge of strategic projects and I simply don't deal with them when I'm working in my home office, because I simply can't motivate myself to do so. Because it's this kind of creative thinking and just, yes, there's just nothing tangible sometimes about projects like that, and I've actually given up trying to do that at home. And then I just work on the most important things, so that the boss is satisfied, because the day-to-day business is done. And the rest I just leave alone, and shift is when I do it in the office." (employee #1, female)
	Structuring and prioritizing tasks	"I have a, I have structured myself in my tasks. So, I work with a Trello board. That means I now also have clearer, I can prioritize for myself what's pending." (employee #8, female)
	Making use of digital communication tools	"Video calls, very important, teams had such a small problem at the beginning, but we adjusted relatively quickly to the new load and you noticed that more and more people then simply turned on their camera. And I think that also had a high impact, to somehow cope with the thing." (employee #19, male)
	Collecting feedback digitally	"Giving feedback anonymously as well. [...] We tried to compensate digitally, and we received some, but not in the usual quality. We did that a) via Webex meetings and b) via anonymous surveys. But that was not the usual feedback as I knew it. I think that has to do with the fact that in open feedback, simply in the open round, that you a) rather dare and b) it comes from one word to the other and pushes each other so that you also talk about it a bit more openly. That way, you only say your basic idea and don't think beyond that. I think that's what describes it that way." (manager #8, male)
	Actively seeking exchange with colleagues	"And if you have a problem like that, if you have the feeling that you have someone who won't open up or something, then of course you always talk to your colleagues anonymously, of course, that's also part of it. And it's also very important for trust. And then you also talk to your people, where you have the feeling that you can have a great conversation with them about it, and that something will come back at that point, feedback. And then you can learn from it. How would the colleague deal with it? For example, I have my assistant next door who, like me, learned to be an industrial foreman at some point, and I always exchange a lot of ideas with him about such things. And that one simply gets these soft topics back into the foreground. We often tend, especially technicians, not to talk much about such things." (manager #5, male)

	Conducting important conversations face-to-face	"At the moment, we have what I would call an interpersonal problem in one team. And that's very difficult to sort out over the phone or by e-mail. And I have just now, before you called, set a personal appointment for us to sit down together next week and sort out this problem." (manager #11, female)
Emotion-focused	Searching for new hobbies	"So, I would say the time gained, in the beginning it was like, I always compare it to New Year's Eve, when you make lots of resolutions. You say: 'I'm going to learn a new language; I'm going to do more sport'. I don't know what other hobbies I had chosen. 'I take more care of my plants ((laughs)), I renovate my house.' And suddenly there were a lot of topics that you had time for, that you usually put on the back burner, because they were topics that had to be dealt with at some point, but not all of them were urgent. And yes, they were very different. At the beginning, of course, people tried to maintain social contacts. That more or less worked, I would say. And then at some point you slipped a little bit into: 'Okay, I'll just look for a new hobby now'. And of course, just like with sport after the New Year's resolution, that lasted for two or three weeks until you realized: 'No, I don't really feel like it' ((laughs))." (employee #17, female)
	Simulating office environment at home	"And that's why, at the beginning, it was good, here now, also nice and quiet, where you say: 'Oh great, nobody's talking in there and you don't hear anything here'. But at some point, it got kind of boring. Until I had the idea: Why don't you turn on the radio? What I normally do when I'm at home because the radio is on for eight hours in the office, but I don't turn it on here at home. Where I say: 'No, I don't need it anymore. I've had that droning all day.' Yes, and now it's the other way round." (employee #13, male)
	Resignation	"Yes, then it's just like that, then you only work five hours, it doesn't matter to me now". (employee #1, female)
	Becoming more relaxed with IT problems	"But we walk much more safely these days because of all the routine we have. So, if I have a technical problem, for example transmission problems, I stay calm and turn off my screen and only do it by phone. It used to be very different, people used to get upset, why can't all this be done? And that has helped me tremendously and I think my colleagues too." (manager #5, male)
	Exercise and sport	"Actually not, because, well, I noticed at the beginning, at the very beginning I didn't do any sport and then I noticed that because I have this long way to go, I also walk a lot, so for example, when I want to go to the office, I first have to walk 15 minutes from my home to the station and I just did

		that every day there and back again every day, simply because I walked this way. And that's when I noticed that I was missing the exercise, because even if you don't actively do sports or anything, you move a lot more, simply because of the journey to the office. And I noticed that I was somehow missing that, which is why I decided to simply integrate this sport into my everyday life." (employee #10, female)
	Meditation	"Psychologically, I was doing quite well, I think. So, it has burdened me somehow, but I have again intensified more meditation. Which led to the fact that I had a good balance, I think. During that time, I also increased the time of meditation. And yes, instead of then somehow meditating the journey to the employer, I think it was a good thing […]." (employee #19, male)
	Conflict mediation	"I'm quite good at this communication thing, because then I do pick up the phone and know how to calm it down." (employee #1, female)
	Not taking things personally	"Well, you only correspond by phone and e-mail, and e-mail in particular can quickly be misunderstood. Then he is quickly annoyed, because somehow there was no 'Please' in the e-mail. And just all the little things. And I personally never take it personally when someone writes me such a snotty e-mail, but I notice time and again, even when I talk to colleagues, that there are total team problems." (employee #1, female)
	Talking to family and friends about feelings	"I tried to talk about it a lot somehow with my partner. And the topic also to, so some topics somehow also to resolve and to create understanding for the other also." (employee #19, male)
	Pursuing hobbies	"Well, I'm outdoors a lot, but I still try to free up the appropriate time for specific sports, as far as possible due to corona. I'm in the garden and do things that are good for me." (manager #12, female)

Note. Categories presented in italics were mentioned by managers only.

Table 4. Overview of Role of Leadership

Category	Role of Leadership	Interview Quotes
Employees' Perspective	Managerial communication	"[My supervisor] just calls in between to find out how I am, how things are going. [...] But this active calling [after online meetings] and asking, 'Can I give you feedback on this? Maybe you can take that into account next time.' I found that very appreciative [...]" (employee #11, female)
	Attitudes and behaviors	"From my point of view, my supervisor rested on that because he refused to work from home all the time. And at times I felt that was a bit unfair. Especially during the time when invoices were due, we realized that my colleague and I both had to go to the office, because otherwise there would have been far too many mistakes. Because of this coordination problem [that the three of us share the office]. And even then, our boss was there instead of saying for two days, 'Well, then I'll just go home.'" (employee #1, female)
	Support provision	"So, my supervisor has taken care of a lot of things that otherwise would have arisen somehow implicitly. Yes, because on the other hand, of course, being the contact person, not only for technical questions, but also, if something goes wrong, but ultimately also for technical things, my monitor doesn't want to connect today." (employee #17, female)
	Staff-care	"Well, my manager regularly asked us whether we could balance, but also separate our work and private lives at home, and how we were doing." (employee #6, female)
	Trust	"I am pretty satisfied [working at home]. Especially because I was also given feedback by my manager in my feedback meeting that I was doing my tasks well and so on. And that's why I rather had the feeling that this also has a lot to do with a lot of trust in me and yes." (employee #18, female)
Managers' Perspective	Staff-care	"Some people you just have to make them follow the work rules. 'Yeah, I know, you're on something right now. You still can't work ten hours a day without justification.' Or in plain language, 'F*** off, please.' ((laughs)) 'Now is the excellent time to clock you out.' That's when you have to protect people from themselves a little bit, too." (manager #2, male)
	Digital leadership	"Because we were always present before and a lot, I'm a manager, I think, who communicates more via symbols or motivation. You can't convey motivation verbally, so you can't do it very well via a Webex or an online meeting, I think. And, since not everyone is always equipped with a camera or has used one, direct contact is missing. So, the facial expressions and gestures of

		employees are missing, and you don't get that back and have a very bad feeling. You can't really assess where you stand and where the team stands." (manager #8, male)
	Communication	"[…] when I communicate with the team, I always turn on my video. The fact that I'm also a manager means that people can see me, that they can see that I'm not sitting there with curlers in my hair ((laughs)) and, I don't know, in a dressing gown. And I also think it's very important for employees to see a face, regardless of whether it's a personnel issue or a management issue. For me, that's trust on the one hand, and the other is just, okay, creating understanding." (manager #1, female)
	Trust	"Otherwise, I don't really like the word, but my leadership style is actually more that you have a lot of trust in each other and that everyone sets it up for themselves the way it works and that, I always like to say, as soon as I don't hear anything, I assume that all is well, but please talk to me if you have a stomachache or if you see an issue somewhere." (manager #14, female)
	Flexibility	"[…] I still see some legal problems there when I get this [teleworkplace contractually] signed. So, on the one hand, things have been neglected in the draft [of telework regulations], on the other hand, the employees are made worse off and now have certain supervisory duties. And I simply don't want them to perform them at all. Because then, if you really take it literally, there's a paper tiger behind it. And I just said, ‘You know what, you're going to get a chair, if you ever stop working here, please bring it back.’" (manager #2, male)
